# Genetic Diversity and Hospital Circulation of Opportunistic Pathogens in COVID-19 ICUs: Whole-Genome Sequencing Data

**DOI:** 10.3390/pathogens15080845

**Published:** 2026-08-13

**Authors:** Svetlana S. Smirnova, Dmitry D. Avdyunin, Yulia S. Stagilskaya, Anastasia A. Kameneva, Tatiana A. Platonova, Nikolai N. Zhuikov, Tarek M. Itani, Aleksandr V. Semenov

**Affiliations:** Federal Scientific Research Institute of Viral Infections «Virome» Rospotrebnadzor, Letnyaya Street, 23, 620030 Yekaterinburg, Russia; avdyunin_dd@niivirom.ru (D.D.A.); stagilskaya_ys@niivirom.ru (Y.S.S.); kameneva_aa@niivirom.ru (A.A.K.); zhuikov_nn@niivirom.ru (N.N.Z.); itani_tm@niivirom.ru (T.M.I.); semenov_av@niivirom.ru (A.V.S.)

**Keywords:** antimicrobial resistance, core genome SNP analysis, healthcare-associated infections, intensive care unit, opportunistic pathogens, personal protective equipment, plasmids, whole-genome sequencing

## Abstract

The COVID-19 pandemic led to a dramatic increase in healthcare-associated infections and antimicrobial resistance, particularly in intensive care units (ICUs). The aim of this study was to provide a comprehensive genomic characterisation of all clinically significant opportunistic pathogens (OPs) isolated from patients and the hospital environment in COVID-19 ICUs, and to use these data to reconstruct transmission pathways, identify reservoirs, and assess the molecular mechanisms of antimicrobial resistance and virulence. Whole-genome sequencing (WGS) was performed on 175 isolates isolated from patients and the hospital environment (including personal protective equipment, PPE) between 2021 and 2023. The species collection included nine OP species. Bioinformatic analysis included multilocus sequence typing, core genome single-nucleotide polymorphism analysis, phylogenetic reconstruction, and in silico detection of resistance and virulence genes and plasmid replicons. High-risk multidrug-resistant (MDR) clones were identified among *Klebsiella pneumoniae*, *Acinetobacter baumannii*, *Escherichia coli*, and *Staphylococcus aureus*. Core genome SNP analysis confirmed direct transmission of *K. pneumoniae* between patients and healthcare worker medical gloves. PPE was identified as a major reservoir, accounting for 68.4% of environmental isolates. The study demonstrates the power of WGS for high-resolution epidemiological surveillance, confirms the critical role of contaminated PPE in nosocomial transmission, and highlights the dominance of internationally spreading MDR clones in COVID-19 ICUs.

## 1. Introduction

The COVID-19 pandemic, caused by the *SARS-CoV-2* virus, posed an unprecedented challenge to healthcare systems worldwide. According to the World Health Organization, by the end of 2023, over 770 million cases and nearly 7 million deaths had been recorded globally. However, beyond the direct consequences of viral infection, the pandemic led to profound and long-term changes in the organisation of medical care, which created favourable conditions for the activation of healthcare-associated infection (HAI) transmission and the spread of antimicrobial resistance (AMR) [1,2].

Of particular concern is the situation in intensive care units (ICUs), which became the epicentre for patients with severe COVID-19 during the pandemic. Extreme workloads on medical staff, resource shortages, the need to rapidly deploy additional beds, and forced deviations from standard infection control protocols created a unique and highly aggressive ecosystem in ICUs [3]. Patients with severe COVID-19, who required invasive mechanical ventilation, received massive immunosuppressive therapy (corticosteroids, interleukin-6 inhibitors) and antimicrobial agents, becoming highly vulnerable to secondary infections by opportunistic pathogens (OPs) [4]. In this context, *SARS-CoV-2*-induced disruption of respiratory epithelium integrity, dysregulation of immune responses, and alterations of the lung microbiome further increased the risk of bacterial superinfection.

A critical factor contributing to the rise in AMR during the pandemic was the irrational use of antibiotics. Faced with the uncertainty of a novel disease and difficulties in the differential diagnosis of viral and bacterial infections, antimicrobials were widely used empirically, often without clear indications. As noted in study involving patients from nine countries, a significant increase in the use of broad-spectrum antibiotics, including meropenem, piperacillin/tazobactam, and azithromycin, was observed during the first 18 months of the pandemic. The most pronounced increase in carbapenem consumption was recorded in countries with high healthcare system burdens, creating strong selective pressure on the microbiota of infectious disease hospitals [5]. According to a systematic review covering studies from Italy, the prevalence of HAIs increased by 9–11% during the pandemic, while the detection rate of multidrug-resistant (MDR) Gram-negative OPs increased in a range from 0.8% to 45.6%, depending on the pathogen and the study population [1].

The scale of the problem is confirmed by data from a retrospective study conducted in Turkey that analysed 1662 respiratory tract samples collected between 2016 and 2024. Comparison of the pre-pandemic and post-pandemic periods revealed a worrying trend: resistance of *Acinetobacter baumannii* to gentamicin increased from 70.9% to 91.3%, to colistin from 3.2% to 12.3%, while resistance of *Klebsiella pneumoniae* to carbapenems (e.g., meropenem: from 50.3% to 77.8%) and colistin (from 23.0% to 62.7%) increased statistically significantly (*p* < 0.001 for both classes) [2]. Moreover, ICU patient mortality in the post-pandemic period showed an increasing trend, from 70.5% to 74.9% (*p* = 0.048), which may be related both to the severity of the underlying disease and to limited treatment options due to high AMR. Notably, despite the overall increase in resistance among most Gram-negative pathogens, the isolation rate of *Escherichia coli* from respiratory samples decreased (from 11.4% to 7.0%, *p* < 0.05), which may reflect changes in the spectrum of nosocomial pathogens during the pandemic [2].

Similarly, the pandemic period was associated with an increased prevalence of methicillin-resistant *Staphylococcus aureus* (MRSA) in hospital settings, driven by high antibiotic pressure and disruptions in infection control practices [6].

At the same time, the impact of the pandemic on AMR was not unidirectional. According to a rapid scoping review, changes in antibiotic use and infection control measures led to heterogeneous effects: while resistance increased in the hospital setting, community and general population settings may have experienced a certain decrease in the prevalence of some resistant strains owing to the introduction of restrictive measures (mask wearing, social distancing, travel restrictions). However, the authors emphasise that most studies had a moderate or high risk of bias, and the analysis of socio-economic determinants and impacts on healthcare systems remained underdeveloped [7].

Particular concern is raised by the role of personal protective equipment (PPE) as a potential reservoir of infection and a factor in its nosocomial transmission. A systematic review evaluating the role of gowns in preventing nosocomial transmission of respiratory viruses showed that the level of PPE contamination reached 77.5%, with the longest preservation of virus infectivity observed on Tyvek suits and plastic gowns [8]. During the pandemic, when staff were forced to work in PPE for extended periods (up to 12 h or more), the risk of contamination of outer surfaces and subsequent pathogen transmission increased significantly. A study conducted in India involving 3098 healthcare workers demonstrated that even with extensive training and observer supervision, 12.7% of staff made mistakes in the sequence of donning PPE, with eye protection being the most frequently omitted element (10.1% of cases) [9]. These data highlight that, even with formal adherence to protocols, significant risks associated with the “human factor” remain.

An important addition to the “human factor” problem is the limited informativeness of traditional microbiological control methods. Routine culture and genotypic typing (e.g., multilocus sequence typing, MLST) often lack sufficient resolution to reliably confirm or refute suspected epidemiological links between isolates, especially under conditions of long-term circulation of genetically related isolates in a closed hospital environment [10,11]. Furthermore, taxonomic complexities and limitations of standard identification methods can lead to incorrect conclusions about isolate relatedness, which in turn hinders timely outbreak recognition and effective infection control measures [12]. The COVID-19 pandemic, placing an enormous burden on healthcare systems, led to a shortage of resources for traditional epidemiological surveillance and infection control auditing, further reducing the effectiveness of standard approaches to HAI prevention. These limitations underscore the need for more accurate and informative molecular analysis methods.

In this context, the use of high-throughput sequencing methods for HAI epidemiological surveillance becomes particularly important. Whole-genome sequencing (WGS) has recently been considered the “gold standard” of molecular epidemiological analysis, allowing not only the identification of the pathogen species and sequence type but also the in silico determination of the complete spectrum of AMR and virulence genes, the detection of plasmid replicons responsible for horizontal gene transfer, and, most importantly, core genome single-nucleotide polymorphism (SNP) analysis to establish the degree of relatedness between isolates with resolution down to a single nucleotide substitution [13]. Data obtained by WGS make it possible to reliably confirm or refute cases of direct microorganism transmission, reconstruct transmission pathways, and assess the contribution of various reservoirs to infection spread [13]. Modern approaches to genomic surveillance, such as the Enhanced Detection System for Healthcare-Associated Transmission, demonstrate not only high efficiency in detecting hidden outbreaks but also cost-effectiveness: the cost of sequencing a single sample in routine surveillance can be less than USD 100 [14].

In the Russian Federation, as in many other countries, the COVID-19 pandemic required the rapid deployment of infectious diseases hospitals with “red” and “green” zones. These institutions became unique model systems for studying the circulation of OPs under conditions of maximal isolation and intensive use of antimicrobial agents. However, despite the urgency of the problem, comprehensive studies covering the entire spectrum of leading OPs (*K. pneumoniae*, *A. baumannii*, *E. coli*, *Staphylococcus aureus*, and others) circulating in such hospitals using WGS have been insufficient to date.

The aim of this study was to provide a comprehensive genomic characterisation of all clinically significant opportunistic pathogens (OPs) isolated from patients and the hospital environment in COVID-19 ICUs, and to use these data to reconstruct transmission pathways, identify reservoirs, and assess the molecular mechanisms of antimicrobial resistance and virulence.

## 2. Materials and Methods

### 2.1. Sampling and Bacterial Isolation

From 2021 to 2023, biological and environmental samples were collected from two large regional infectious diseases hospitals in the Sverdlovsk region, Russian Federation, repurposed for treating patients with severe COVID-19. One of the hospitals had a 440-bed capacity, including a 12-bed ICU; the other hospital had an ICU of comparable size (approximately 12 beds). Sampling was performed exclusively within the ICUs of both hospitals, which were thus representative of the larger, strained healthcare system responding to the severe wave of the pandemic.

The sampling scheme, including the selection of 20 high-contact sampling points grouped into three blocks (PPE of medical staff, patient care environment—PCE, and general hospital points—GHP), was developed by the authors for simultaneous assessment of viral and bacterial contamination [15]. Sterile cotton swabs pre-moistened with 0.1% peptone water supplemented with 0.5% sodium thiosulfate were used to collect surface samples at 4 h intervals over a 72 h period. This method was described in detail in our previous work [16] and was applied here without modification, with the important extension that the current study incorporates the earlier *A. baumannii* data and adds a second hospital, a longer time frame (2021–2023), and a broader collection of 175 isolates covering nine species (see Section 3.1).

Clinical specimens—comprising 28 sputum samples, 14 oropharyngeal swabs, one urine sample, and one urethral swab—were collected from ICU patients. All patients had been admitted with viral pneumonia due to SARS-CoV-2; bacterial isolates recovered from these specimens represent colonising or co-infecting microbiota rather than primary bacterial infections. Sampling and processing followed the national guidelines MUK 4.2.2942-11 [17].

A complete list of isolates with sample IDs and sampling points is provided in Appendix A.

### 2.2. DNA Extraction and Whole-Genome Sequencing

DNA extraction and library preparation for the isolates sequenced on the Illumina MiSeq platform were performed as described previously [16]. The remaining isolates (comprising the majority of *K. pneumoniae*, *E. faecalis*, *E. faecium*, *P. aeruginosa*, *P. mirabilis*, and four *A. baumannii* isolates) were sequenced on the MGI DNBSEQ-G50 platform. For these isolates, DNA extraction was performed using the “MAGNO-sorb-M” kit (FBIS CRIE, Moscow, Russia) according to the manufacturer’s instructions, using an Auto-Pure 96 station (Allsheng, Hangzhou, China). DNA concentration was measured with a Fluo200 fluorometer and the QuDye dsDNA HS assay kit (Lumiprobe, Moscow, Russia). Library preparation was carried out following the MGIEasy Fast FS Library Prep Set User Manual (v2.0). Briefly, 100–500 ng of genomic DNA was fragmented with Fast FS II enzyme (MGI, Shenzhen, China) for 25 min at 30 °C, followed by a 0.8× size selection using MGI Easy DNA Clean Beads. Universal UDB adapters were ligated to the fragments, and adapter-ligated products were purified with a 0.8× bead cleanup. Indexing PCR was performed with limited cycles (5–8 cycles) using UDB primers. The PCR product was purified with 0.9× beads. Library quality was assessed using a Fluo200 fluorometer and a Qsep1 capillary electrophoresis system (target fragment size ~300–500 bp). DNA nanoballs (DNBs) were prepared using the DNBSEQ One-step DNB Make Reagent Kit V2.0 (MGI, Shenzhen, China) according to the manufacturer’s protocol. Libraries were pooled in equimolar amounts, and the final pool was quantified with the QuDye dsDNA HS kit. Sequencing was performed on a DNBSEQ-G50 instrument using an FCL flow cell and the PE150 reagent kit (300 cycles, paired-end 2 × 150 bp).

### 2.3. Bioinformatic Analysis

Open-source software was used to analyze the data. Raw sequencing reads were quality-controlled with FastQC 0.12. For genome assembly, paired-end reads were processed with SPAdes 4.2.0 [18] through the Galaxy platform. Species confirmation and contamination screening were performed using FCS-GX 0.5.5 [19]. Assemblies were then cleaned of adapter sequences via NCBI VecScreen (FCS Adaptor 0.5.5), and contigs were filtered by length using fastp 1.3.3 [20].

Sequence typing was carried out using the PubMLST scheme (https://pubmlst.org/, accessed on 4 May 2026) and the Galaxy-based MLST tool 2.22.0.

The analysis of assembled genomes was performed using several bioinformatic tools and databases. AMR genes were identified using ResFinder 4.6.0 and AMRFinderPlus 3.12.8 [21,22]. Virulence genes were detected using the ABRicate utility 1.4.0 [23] with the VFDB database. Plasmid replicons were determined using PlasmidFinder 2.1.6 [24]. All searches were conducted with thresholds of ≥90% nucleotide identity and ≥60% coverage.

For phylogenetic analysis, genome annotation was performed using Prokka 1.14.6 [25]. The core genome alignment was constructed with Roary 3.13.0 [26] using a 90% threshold for gene presence (rather than the default 99%) to retain greater genetic diversity. Non-informative columns (e.g., positions containing only undetermined values, which were treated as missing data as identified by RAxML 8.2.12 [27]) were removed using a custom Python script (version 3.14.3). Maximum-likelihood phylogenetic trees were reconstructed with IQ-TREE 2.0.7 [28] using 1000 bootstrap replicates. Reference genomes of the corresponding species and relevant sequence types were obtained from PubMLST (https://pubmlst.org/, accessed on 5 May 2026), NCBI (https://www.ncbi.nlm.nih.gov/, accessed on 6 May 2026), and the VGARus (developed and maintained by the Central Research Institute of Epidemiology of Rospotrebnadzor, Moscow, Russia) database and included in the tree reconstruction. The VGARus database is a restricted-access resource for genomic surveillance in the Russian Federation.

To assess recent transmission events, a core genome SNP analysis was performed on isolates that showed similarity in their resistance and virulence profiles. The core genome alignment generated by Roary was used to calculate pairwise SNP distances with the SNP distance matrix tool (version 0.8.2) [29]. Isolates differing by fewer than 15 SNPs were considered genetically linked, which is consistent with established criteria for recent nosocomial transmission.

Interpretation of SNP distances is highly species-specific: the rate of mutation accumulation, core genome size, and the time interval between sampling affect the choice of threshold. In epidemiological investigations, it is recommended to combine genomic data with classical epidemiological methods and to use literature-derived thresholds as a guide (e.g., ≤6–10 SNPs for recent transmission in *Enterobacteriaceae* and ≤15–20 SNPs for non-fermentative bacteria such as *A. baumannii*) [30,31,32,33].

Descriptive statistics (percentages) were calculated using Microsoft Excel.

## 3. Results

### 3.1. Genetic Characterisation of Hospital Pathogens

From 2021 to 2023, a total of 175 OP isolates were obtained in the ICUs of infectious diseases hospitals treating patients with COVID-19. Of these, 36 isolates originated from ICU patients, and 139 were collected from the environment ICU. Among the environmental isolates, 76 were recovered from the surface of PPE, 52 from the PCE, and 11 from GHP.

The species composition included nine species: *A. baumannii* (*n* = 60; 34.3%), *K. pneumoniae* (*n* = 53; 30.3%), *S. aureus* (*n* = 20; 11.4%), *Enterococcus faecalis* (*n* = 20; 11.4%), *E. coli* (*n* = 10; 5.7%), *Proteus mirabilis* (*n* = 5; 2.9%), *Pseudomonas aeruginosa* (*n* = 4; 2.3%), *Enterococcus faecium* (*n* = 2; 1.1%), and *Klebsiella variicola* (*n* = 1; 0.6%). *K. pneumoniae* and *A. baumannii* played a dominant role in the OP structure both among patients and in the hospital environment.

The key genetic characteristics of the main OP species—including dominant clones, resistance determinants, virulence factors, and plasmid replicons—are summarised in Table 1. This table provides a quick overview of the most clinically significant features for each species, allowing comparison across the nine pathogens identified in this study. Detailed descriptions of typing, antimicrobial resistance profiles, virulence gene and plasmid replicons repertoires for each species are provided in Section 3.1.1, Section 3.1.2, Section 3.1.3, Section 3.1.4, Section 3.1.5, Section 3.1.6, Section 3.1.7 and Section 3.1.8, while the complete datasets are available in Supplementary Tables S1–S3.

#### 3.1.1. *Acinetobacter baumannii* Isolates

Of the 60 *A. baumannii* isolates, nine (15.0%) were obtained from clinical specimens of patients, and 51 (85.0%) from the environment, of which 23 (45.1%) came from PPE.

**Figure 1:**
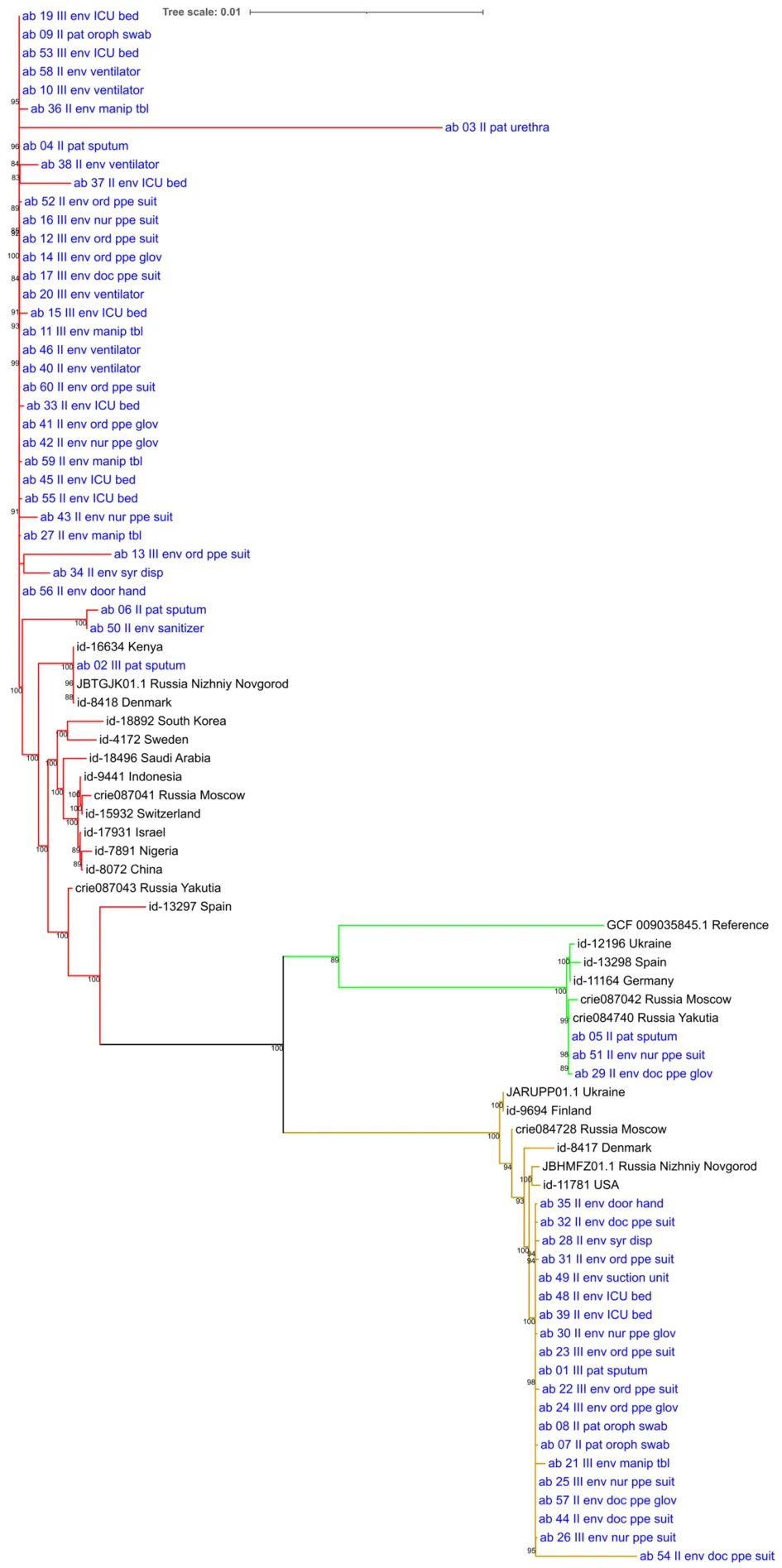
Maximum-likelihood phylogenetic tree of *A. baumannii* clinical isolates and reference sequences from the PubMLST, NCBI, VGARus databases. Coloured clades: red (ST2), light green (ST19), and golden yellow (ST78). Blue: study isolates; Black: PubMLST, NCBI, VGARus databases; Green: GenBank species reference.

Typing and Phylogeny. MLST identified three sequence types (STs) among the 60 *A. baumannii* isolates: ST2 (*n* = 24; 40.0%), ST78 (*n* = 17; 28.3%), and ST19 (*n* = 2; 3.3%). Seventeen isolates (30.4%) were non-defined. Phylogenetic analysis based on a soft-core genome alignment (Figure 1) distributed all studied isolates into the three STs—ST19, ST78, and ST2. The ST19 isolates (including one non-defined isolate, ab_29) formed a compact cluster together with Russian reference sequences (crie084740, crie087042), separating from European isolates. Within ST78, two main subclades emerged: one consisted exclusively of isolates from the second sampling phase, while the other included a mixed group from the second and third phases, suggesting the simultaneous circulation of two distinct subpopulations. ST2 appeared to be the most heterogeneous. In addition to several separate lineages (basal isolate ab_09, a branch with isolates from Denmark, Kenya, and Nizhny Novgorod, as well as the pair ab_50+ab_06 and isolate ab_56), a large cluster was identified within which two genetically distinct subclades could be distinguished. Both subclades contained isolates from the second and third sampling phases, indicating long-term parallel circulation of two independent ST2 lineages within the hospital. The non-defined isolates were distributed among all three STs, confirming that no other STs were present in the hospital during the study period.

AMR. All *A. baumannii* isolates carried genes conferring resistance to aminoglycosides and β-lactams. ST2 was characterised by the profile: *armA*, *aph(6)*-*Id*, *aph(3′)*-*Ia*, *aph(3″)*-*Ib*, *bla*_OXA-23_, *msr(E)*, *mph(E)*. The carbapenemase gene *bla*_OXA-23_ was present in 100% of ST2 isolates. ST78 was characterised by the presence of *armA*, *bla*_OXA-72_, *bla*_OXA-90_, *msr(E)*, *mph(E)*, while *bla*_OXA-23_ was absent. ST19 carried *aph*(3′)-*VIa*, *bla*_OXA-69_, *bla*_ADC-25_, *catA1*, *sul2*, and *tet(B)*. The resistance profile of non-defined isolates was similar to that of ST2, suggesting genetic relatedness.

Virulence. The virulence gene repertoire of *A. baumannii* included the *csu* operon (adhesins), bas genes (biofilm/acinetobactin), the type VI secretion system (T6SS), outer membrane proteins (*ompA*), the regulatory system *pmrB*, and multiple efflux pumps (*ade*, *abe*).

#### 3.1.2. *Klebsiella pneumoniae* Isolates

Fifteen isolates (28.3%) were obtained from clinical specimens of ICU patients, and 38 (71.7%) from the ICU environment, of which 15 (39.5%) came from PPE.

Typing and Phylogeny. A total of 53 isolates were obtained. The dominant clone belonged to ST512 (*n* = 25; 47.2% of all isolates). Other STs detected included ST101, ST1190, ST15, ST307, ST395, as well as non-defined isolates. Intra-hospital circulation of ST512 was confirmed: isolates were recovered from nine patients and from the hospital environment (seven from PPE, nine from PCE).

**Figure 2:**
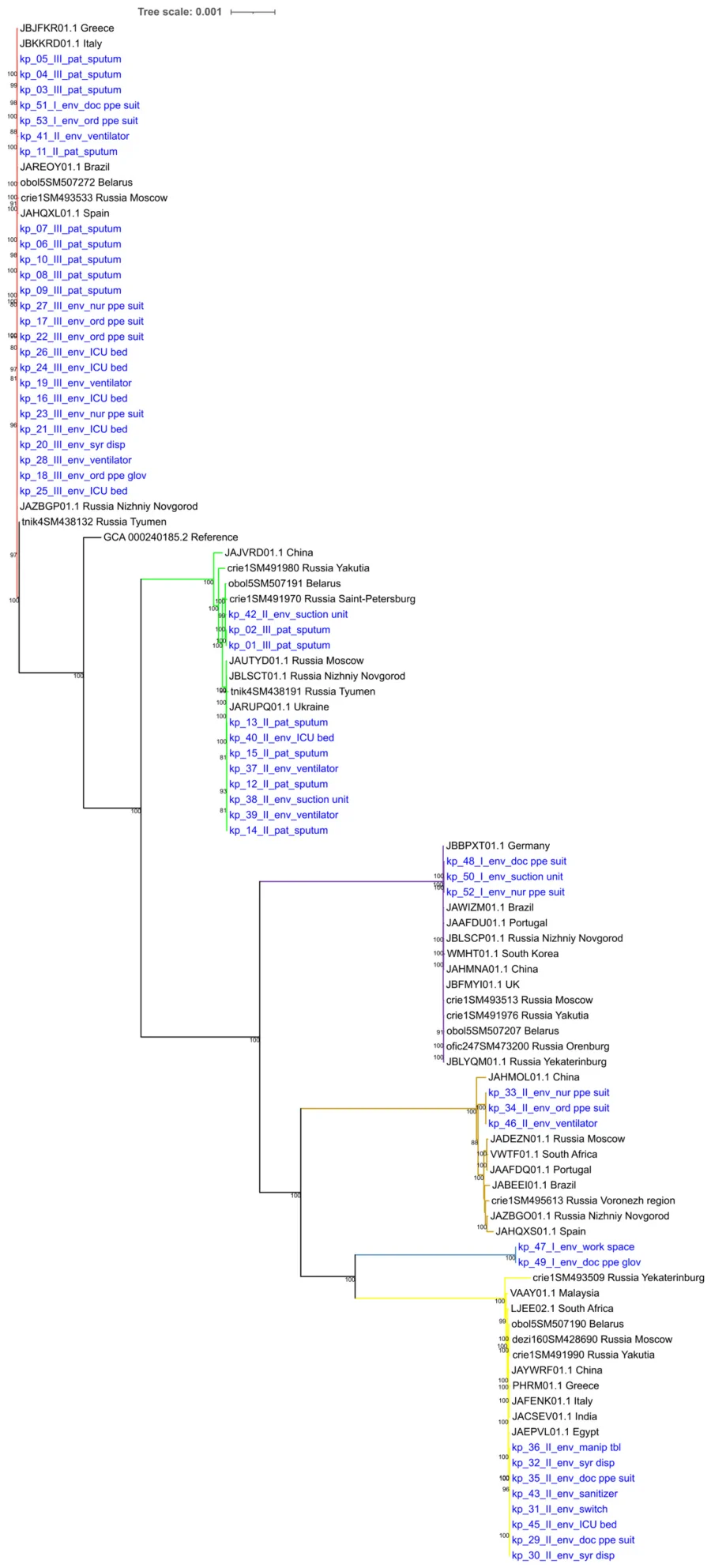
Maximum-likelihood phylogenetic tree of *K. pneumoniae* clinical isolates and reference sequences from the PubMLST, NCBI, VGARus databases. Coloured clades: red (ST512), light green (ST395), purple (ST307), golden yellow (STST15), light blue (ST1190) and yellow (ST101). Blue: study isolates; Black: PubMLST, NCBI, VGARus databases; Green: GenBank species reference.

Phylogenetic analysis based on a soft-core genome alignment (Figure 2) distributed the isolates into six STs. The ST101 branch was distinct, with two parallel sublines; all ST101 isolates belonged exclusively to the second sampling phase and showed relatedness to an Egyptian reference genome. ST1190 was extremely rare (two isolates), and no closely related reference sequences were found in the databases; no mixing of isolates from different phases was observed. ST15 and ST307 also formed separate clusters without mixing of phases, and ST307 did not show clear affinity to any geographic group (Russian, European, etc.). The ST395 branch split into two subclades: the first contained three isolates (one from the second phase and two from the third) and clustered with Russian and Belarusian isolates; the second subclade consisted exclusively of non-defined isolates from the second phase, close to Russian–Ukrainian references. This structure suggests that ST395 is endemic to the study region. The largest and most heterogeneous group was ST512, within which two major clusters formed: one comprised exclusively isolates from the third phase, while the other (smaller) contained isolates from the first, second and third phases simultaneously. Notably, in this smaller subcluster, the first-phase isolates originated from the first hospital, while the related second-phase isolates came from the second hospital.

AMR. The dominant clone ST512 (*n* = 25) exhibited a MDR profile including the carbapenemase gene *bla*_KPC-3_ (carbapenems, 25/25), the chromosomal colistin resistance mutation *pmrB*(R256G) (24/25), the fluoroquinolone resistance determinants *gyrA*(S83I) (24/25) and *oqxAB* (25/25), the biocide resistance gene *qacEdelta1* (24/25), and the fosfomycin resistance gene *fosA* (25/25).

The other sequence types (ST15, ST101, ST307, ST395 and ST1190) were also MDR but differed markedly from ST512. None of them carried carbapenemase genes (*bla*_KPC-3_, *bla*_NDM-1_, *bla*_OXA-48_), the colistin resistance mutation *pmrB*(R256G), or the biocide resistance gene *qacEdelta1*. Instead, their β-lactam resistance was mediated by extended-spectrum β-lactamase (ESBL) genes, predominantly *bla*_CTX-M-15_, often in combination with *bla*_OXA-1_, *bla*_SHV-28_, *bla*_TEM-1_ or *bla*_SHV-1_. All these isolates possessed various aminoglycoside resistance genes (*aac(6′)-Ib-cr5*, *aph(3′)-Ia*, *aph(3″)-Ib*, *aph(6)-Id*, *aadA1*, *aadA2*), fluoroquinolone resistance determinants (mutations in *gyrA* and *parC*, and/or *qnrS1*, *qnrB*), tetracycline resistance (*tet(A)*), phenicol resistance (*catA1*, *catA2*, *catB3*), sulphonamide resistance (*sul1*, *sul2*), and macrolide resistance (*erm(B)*, *mph(A)*). Fosfomycin resistance (*fosA*) was detected only in ST395. The resistance profiles of ST101 and ST307 were similar to each other, while ST395 additionally carried *bla*_SHV-1_, *erm(B)*, *mph(A)*, *catA1*, *qnrB* and *fosA*. ST1190 harboured *bla*_CTX-M-15_, *aph(3′)-Ia*, *aph(3″)-Ib*, *aph(6)-Id*, *catA1*, *oqxB19*, *sul1*, *sul2*, and *qnrS1*. Thus, genetically diverse *K. pneumoniae* lineages with different resistance mechanisms co-circulated in the hospital, and the carbapenemase, colistin and biocide resistance markers were exclusive to the dominant ST512 clone.

Virulence. All *K. pneumoniae* isolates carried the *ecp* adhesin operon and *ompA*. Siderophore profiles varied: ST512 and some ST395 isolates carried the yersiniabactin system (*fyuA*, *ybt*, *irp*) together with enterobactin (*ent*, *fepC*), while other STs carried enterobactin only or enterobactin with aerobactin. No classical toxin genes were detected.

A wide diversity of plasmid replicons was detected among the isolates. The most frequent were IncFIB(K) (71.7% of isolates), IncFII(K) (67.9%), IncRNAI (64.2%), IncX3 (45.3%) and IncHI1B (34.0%).

In addition, a single isolate of *K. variicola* ST4101 was recovered from PPE. It carried resistance genes for aminoglycosides (*aph(3″)-Ib*, *aph(6)-Id*), β-lactams (*bla*_LEN-2_), fluoroquinolones (*oqxA*, *oqxB*), fosfomycin (*fosA*), as well as the plasmid replicons IncFIB(K), IncFIB(pKPHS1), IncFII(K) and Col(pHAD28).

#### 3.1.3. *Escherichia coli* Isolates

Two isolates (20.0%) were obtained from clinical specimens of ICU patients, and eight (80.0%) from the ICU environment, of which five (63.0%) came from PPE.

**Figure 3:**
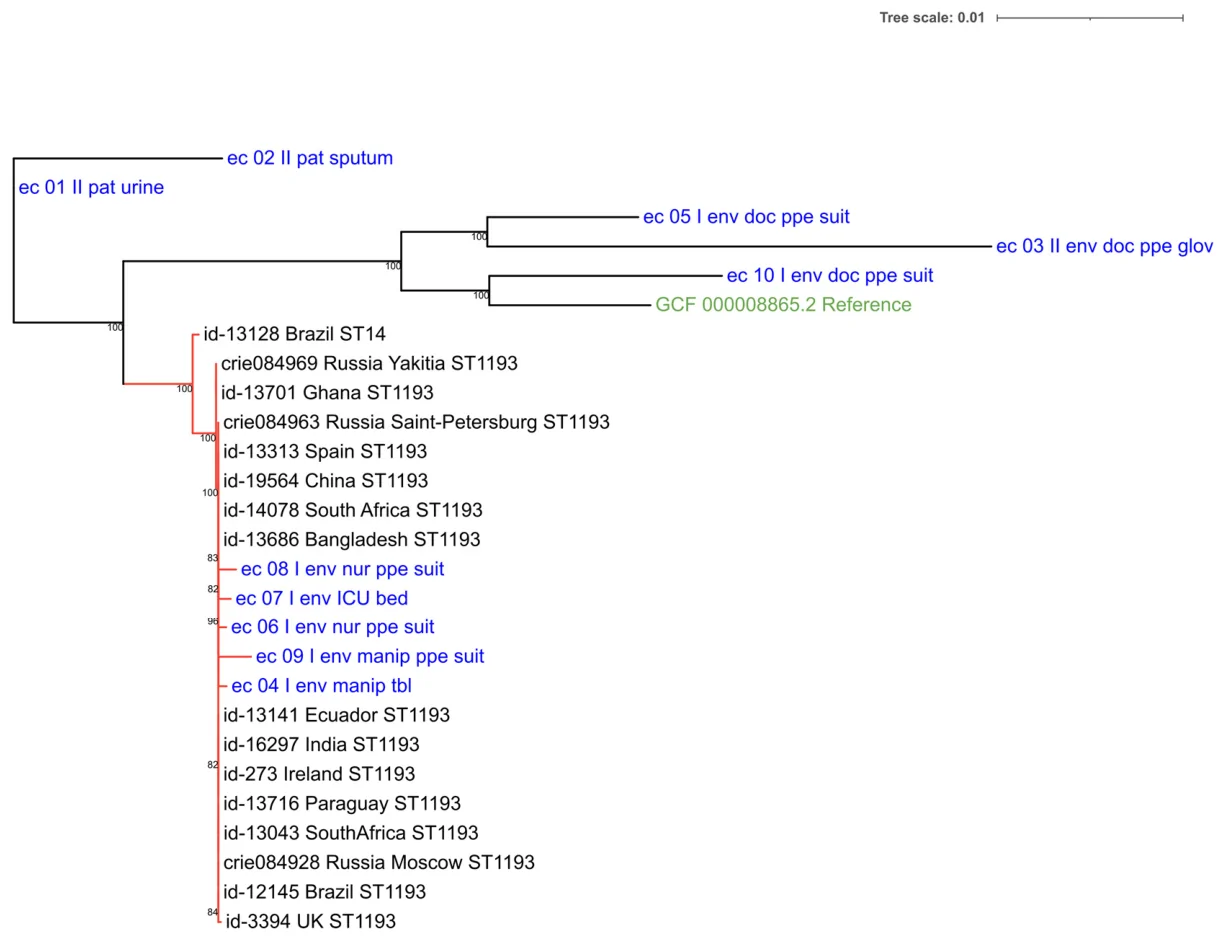
Maximum-likelihood phylogenetic tree of *E. coli* clinical isolates and reference sequences from the PubMLST, NCBI, VGARus databases. Coloured clades: red (CC14). Blue: study isolates; Black: PubMLST, VGARus databases; Green: GenBank species reference.

Typing and Phylogeny. Although none of our *E. coli* isolates (*n* = 10) could be typed by standard MLST schemes (probably due to technical limitations or loss of target genes), phylogenetic analysis based on a soft-core genome alignment (Figure 3) allowed the assessment of their genetic relatedness. Most environmental isolates (first sampling phase) formed a compact subcluster within the ST1193 lineage (Clonal Complex 14, CC14) with high bootstrap support (96–100), indicating their assignment to this ST. Other isolates (including patient samples) showed mixing of phases: some grouped with reference sequences, while others formed separate branches. Thus, the tree structure suggests the circulation of at least two genetically distinct *E. coli* lineages in the hospital, one of which (ST1193) was represented exclusively by first-phase isolates from the hospital environment.

AMR. In silico analysis revealed resistance determinants against eight classes of antimicrobials: aminoglycosides (*aph(3″)-Ib*, *aph(6)-Id*, *aadA25*), β-lactams (*bla*_CTX-M-15_, *bla*_TEM_, *bla*_TEM-1_, *bla*_TEMp_(C32T)), quinolones (*qnrS1*, mutations *parC*(80I), *gyrA*(D87N), *gyrA*(S83L)), antifolates (sul2), fosfomycins (*uhpT*(E350Q), *glpT*(E448K), *fosA*, *cyaA*(S352T)), colistin (*pmrB*(E123D)), nitrofurans (*nfsB*(W94STOP)), and multidrug resistance (*marR*(S3N)). This gene group was the most prevalent, indicating a high adaptive potential of the population.

The *bla*_CTX-M-15_ gene was detected in 22% of isolates, mainly among ST1193. All CC14 isolates carried *marR*(S3N); *bla*_TEM_ group genes were found in 80% of CC14 isolates. Among isolates from patient care surfaces, 100% carried multidrug resistance genes, whereas isolates from PPE showed greater diversity of resistance determinants (16 out of 21 unique genes).

Virulence. The most frequently detected genes were associated with adhesion (*fimH*—100%, *yehA*/*B*/*C*/*D*—up to 90%), invasion (*ibeA*—33%) and immune evasion (*traT*—78%). Hypothetical ST1193 isolates carried a full set of adhesion genes. The hypothetical ST1380 isolate harboured the rare vat gene encoding a serine protease autotransporter characteristic of some uropathogenic *E. coli*. The clinical isolate from a patient contained the highest number of virulence genes (15).

Plasmid replicons were found in 100% of isolates; the dominant types were IncFIA, IncFIB and ColBS512.

#### 3.1.4. *Staphylococcus aureus* Isolates

Three isolates (15.0%) were obtained from clinical specimens of ICU patients, and 17 (85.0%) from the ICU environment, of which 14 (82.4%) came from PPE.

Typing and Phylogeny. Among the 20 *S. aureus* isolates, a full ST could be assigned for six (30%): ST22 (*n* = 1), ST97 (*n* = 1), ST5727 (*n* = 3), ST7614 (*n* = 1). For 14 (70%) of isolates, full typing was impossible due to the absence of alleles for one or more housekeeping genes; however, for seven of them, assignment to CCs was possible. Four CCs circulated in the ICU: CC15 (30%, *n* = 6), CC8 (15%, *n* = 3), CC22 (10%, *n* = 2), and CC97 (10%, *n* = 2). The remaining seven isolates could not be assigned to any specific ST or CC—non-defined.

**Figure 4:**
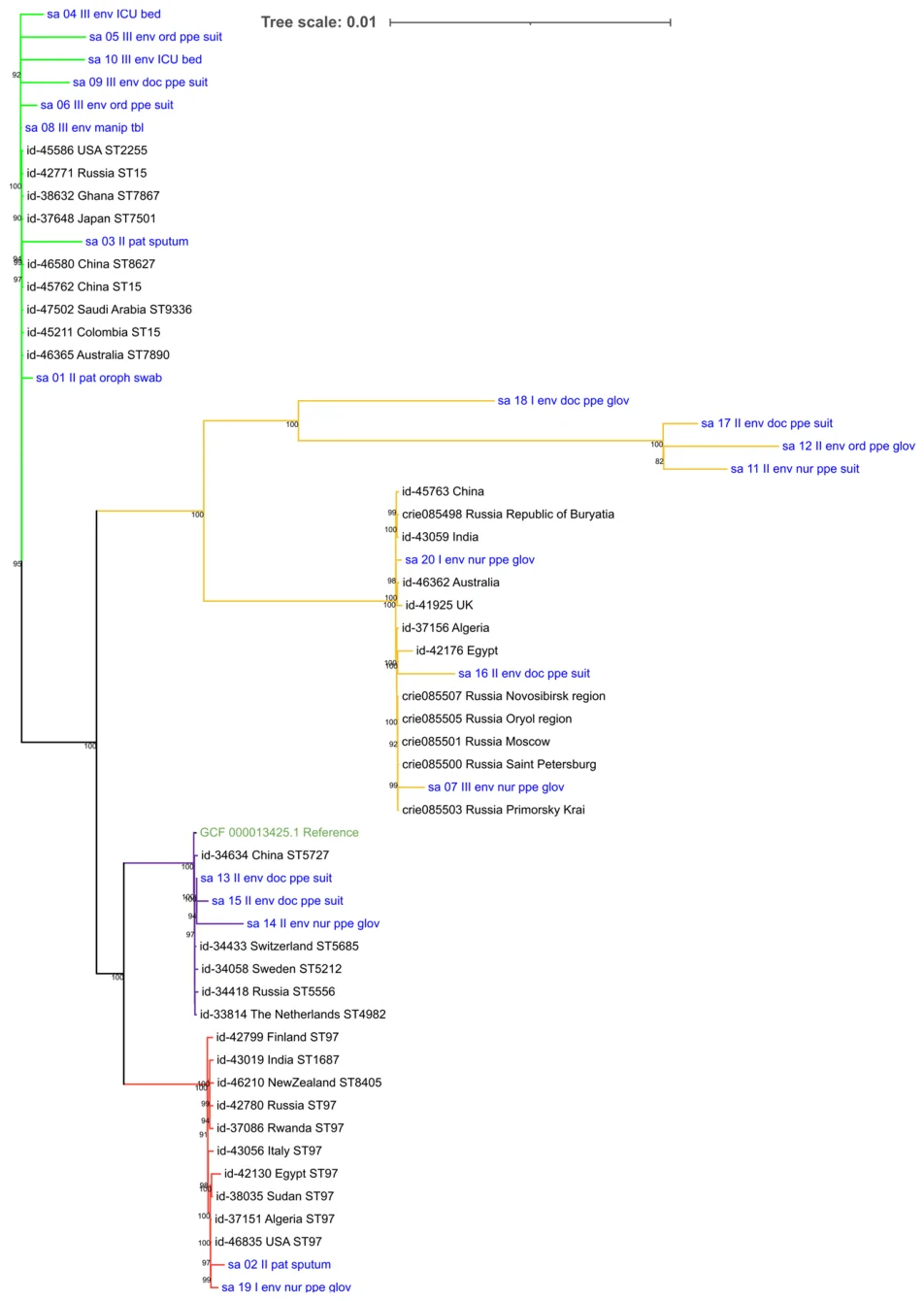
Maximum-likelihood phylogenetic tree of *S. aureus* clinical isolates and reference sequences from the PubMLST, NCBI, VGARus databases. Coloured clades: red (CC97), light green (CC15), purple (CC8), golden yellow (CC22). Blue: study isolates; Black: PubMLST, VGARus databases; Green: GenBank species reference.

Despite the fact that a significant proportion of our *S. aureus* isolates could not be typed by standard schemes, phylogenetic analysis based on a soft-core genome alignment (Figure 4) reliably assigned them to four clonal complexes—CC8, CC15, CC22 and CC97. CC97 isolates formed a single cluster with isolates from the USA and Algeria. Within CC8, our isolates formed a sister group to European sequences from Russia, Sweden, the Netherlands and Switzerland. CC22 showed the greatest heterogeneity: one lineage clustered with Russian genomes, another with an Egyptian isolate, a third with isolates from Australia and the United Kingdom, and a separate group of non-defined isolates (three from the second phase and one basal isolate from the first phase) also emerged. Within CC15, our isolates were distributed among several subclades without clear mixing by phase in closely related nodes; some grouped with Australian, Chinese, Colombian and Saudi isolates, while others formed independent local branches. Thus, the tree structure confirms the circulation of four *S. aureus* CCs in the hospital, with the greatest diversity among our isolates observed in CC15.

AMR. Resistance determinants against nine classes of antimicrobials were identified. The most common were β-lactam resistance genes: *blaZ* (65%), *blaI* (60%), *blaR1* (55%), as well as tetracycline resistance (*tet*(38)—95%). Methicillin-resistant *S. aureus* (MRSA) carrying *mecA* and *mecR1* were detected in 100% of CC22 isolates and in 14.3% of non-defined isolates. All CC8 and CC15 isolates carried *fosB* (fosfomycin resistance). CC22 showed 100% presence of *dfrB*(A135T) (trimethoprim resistance). The *fusA*(A655V) mutation (fusidic acid resistance) was found in 50% of CC97 isolates.

Isolates from PPE displayed the greatest diversity of resistance determinants, whereas isolates from PCE carried β-lactam resistance genes in 100% of cases.

Virulence. Genes encoding exoenzymes (*aur*—90%, *splA*/*B*/*E*—60–70%), host immune evasion factors (*sak*—55%, *scn*—75%), and toxins (*hlgA*/*B*/*C*—90%, *lukD*/*E*—50–60%) were widely distributed. Enterotoxins (*sea*, *seb*, *seg*, *sei*, *sem*, *sen*, *seo*, *seu*) were detected in 10–30% of isolates, mainly among CC22 and some non-defined isolates. The toxic shock syndrome toxin gene (*tst*) was found in 5% of isolates.

Plasmid replicons were detected in 85% of isolates; the most frequent were rep16 and rep5a.

#### 3.1.5. *Enterococcus faecalis* Isolates

Three isolates (15.0%) were obtained from clinical specimens of ICU patients, and 17 (85.0%) from the ICU environment, of which 13 (76.5%) came from PPE.

Typing. A total of 20 isolates were typed by MLST, identifying nine STs: ST30 (*n* = 5), ST40 (*n* = 3), ST387 (*n* = 2), ST832 (*n* = 2), ST25, ST44, ST179, ST228 and ST2080 (one each), and three non-defined isolates.

AMR. The most frequent genes were *lsa(A)* (85%), *dfrE* (80%), *emeA* (80%), *efrA* and *efrB* (each 75%), *tetM* (60%), *erm(B)* (45%). Aminoglycoside resistance genes (*aad(6)*, *aph(3′)-IIIa*, *sat*-4) were found in 40% of isolates, and *aac(6′)-Ie*-*aph(2″)-Ia* in 20%.

Virulence. Nearly all isolates carried genes encoding adhesion factors (*ebpA*, *ebpB*, *ebpC*, *srtC*, *efaA*, *prgB*/*asc10*), the two-component regulatory system (*fsrABC*), gelatinase (*gelE*), serine protease (*sprE*), as well as capsule biosynthesis genes (*cpsA*–*K*). Cytolysin operon genes (*cyl*) were detected in 45% of isolates. The aggregation substance gene *asa1* was found in 15% of isolates, and the adhesin *bopD* in 85%.

Plasmid replicons were detected in 70% of isolates; the most frequent were rep9b and repUS43.

#### 3.1.6. *Enterococcus faecium* Isolates

Isolates were obtained exclusively from the ICU environment, with two isolates (100%) detected on PPE; no clinical isolates from patients were registered.

Typing. The two obtained isolates belonged to ST52.

AMR. Both isolates carried *aac(6′)-I* (aminoglycosides), *msr(C)* (macrolides), and *eat(A)*(T450I). No other resistance determinants were found.

Virulence. No virulence genes were detected.

Both isolates carried identical plasmid replicons: repUS15, rep1, rep14b.

#### 3.1.7. *Proteus mirabilis* Isolates

Two isolates (40.0%) were obtained from clinical specimens of ICU patients, and three (60.0%) from the ICU environment, of which two (66.7%) came from PPE.

Typing. Five isolates were obtained, belonging to two STs: ST269 (*n* = 3) and ST234 (*n* = 2).

AMR. All isolates exhibited multidrug resistance in silico. β-Lactam resistance genes detected: *bla*_CTX-M-3_ (in three isolates), *bla*_CTX-M-14_ (in two), *bla*_TEM-1_ (in four). Additional resistance genes were found against aminoglycosides (*aadA2*, *aph(6)-Id*, *aph(3″)-Ib*, *rmtB*, *aac(3)-IIe*, *ant(3″)-IIa*), sulphonamides (*sul1*, *sul2*), tetracyclines (*tet(J)*), chloramphenicol (*catI*, *catA4*), trimethoprim (*dfrA1*, *dfrA12*). The streptothricin resistance gene *sat-1* was present in four isolates.

Virulence. All isolates carried genes encoding adhesion factors (*mrpA*, *pmfA*, *fimA*, *fimH*), secretion systems (T6SS: *hcp*, *vgrG*, *clpV1*), the urease operon (*ureA–G*), flagellar genes, regulatory systems (*crp*, *cpxAR*, *phoPQ*, *rcsAB*), as well as iron-siderophore transport genes (*fyuA*, *hemR*, *hmuTUV*, *exbD*). Some isolates harboured toxin genes (*hpmA*, *tdhA*, *hlyB*/*tolC*).

Plasmid replicons. All isolates carried the IncC replicon. One isolate (ST234) additionally harboured the Col3M replicon.

#### 3.1.8. *Pseudomonas aeruginosa* Isolates

Two isolates (50.0%) were obtained from clinical specimens of ICU patients, and two (50.0%) from the ICU environment, of which one (50.0%) came from PPE.

Typing. Four isolates were obtained, belonging to three STs: ST654 (*n* = 2), ST235 (*n* = 1), ST549 (*n* = 1).

AMR. The ST235 isolates carried aminoglycoside resistance genes (*aph(3′)-IIb*, *aph(3′)-XV*, *aac(6′)-Ib4*, *aadA6*), the carbapenemase *bla*_GES-1_, oxacillinases *bla*_OXA-488_, *bla*_OXA-50_, *bla*_PDC-35_ and *bla*_PDC-195_, the mutation *oprD*(W417STOP), phenicol resistance genes (*catB7*, *floR2*), tetracycline resistance genes (*tetR(G)*, *tet(G)*), *fosA*, *gyrA*(T83I), *sul1*, and *qacEdelta1*. While ST654 isolates’ profile included *bla*_VIM-2_, *bla*_OXA-396_, *bla*_PDC-3_, *bla*_PDC-216_, *strA/strB*, *aph(3′)-IIb*, *aph(3″)-Ib*, *aph(6)-Id*, *aph(3′)-VI*, *catB7*, *floR2*, *tet(A)*, *tet(G)*, *fosA*, *parC*(*S87L*), *gyrA*(T83I), *sul1*. ST549: the smallest set—*aph(3′)-IIb*, *bla*_OXA-50_, *bla*_PDC-1_, *bla*_PDC-212_, *catB7*, *fosA*, *crpP*.

Virulence. All isolates carried an extensive set of virulence genes, including regulators (*lasI/R*, *rhlI*, *exsA-E*, *algU*, *mucA-D*), adhesion factors (pil genes), secretion systems (T3SS, T6SS), exotoxins (*toxA*, *exoT*, *exoS*, *exoY*), pigment biosynthesis (*phz* operon), siderophores (*pvd*, *pch*, *pvc*), and flagellar genes.

Plasmid replicons were not detected in any of the *P. aeruginosa* isolates.

### 3.2. Role of the Hospital Environment and PPE in Nosocomial Transmission of Infection

#### Contaminated Sites

**Figure 5:**
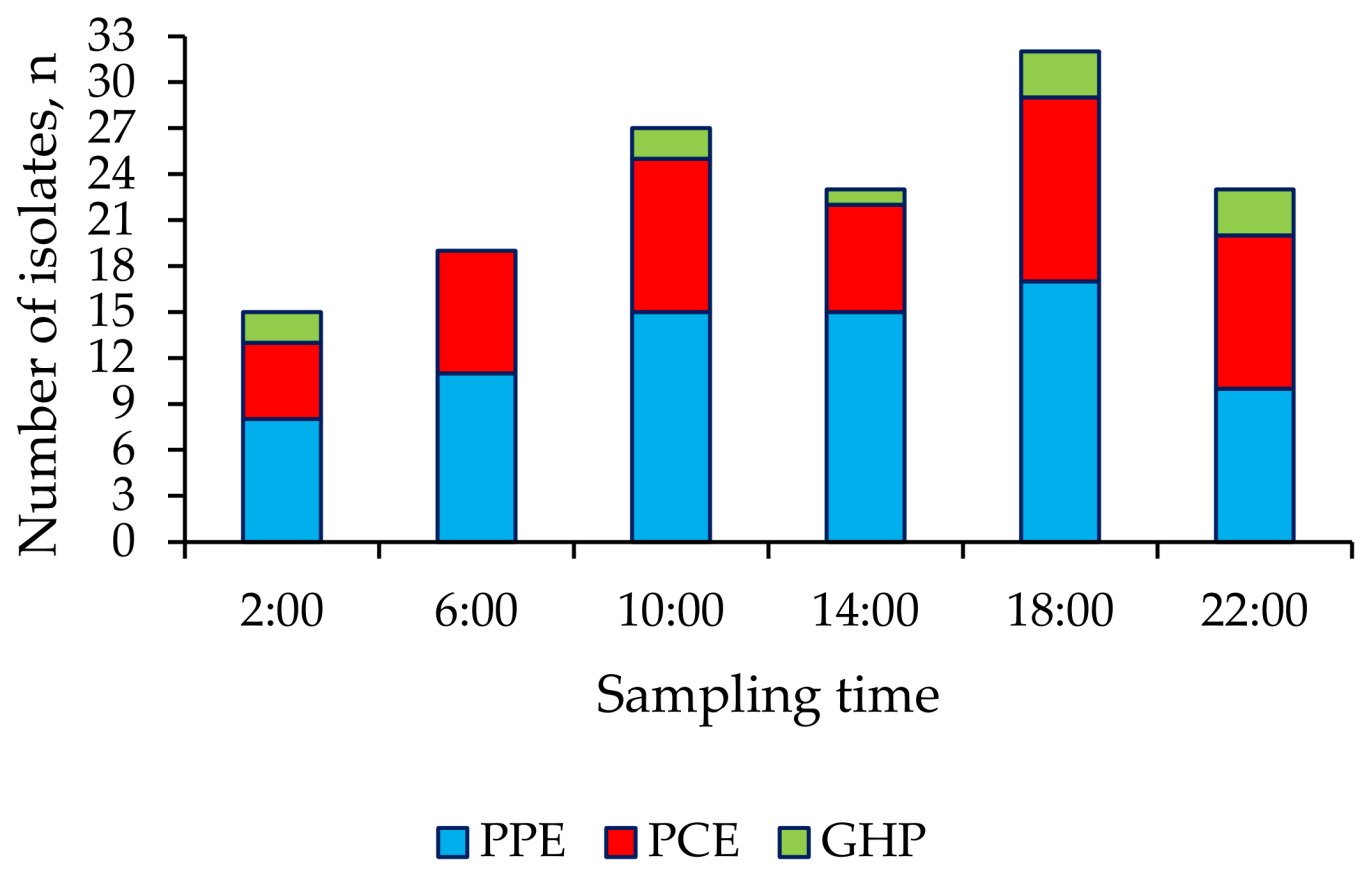
Temporal dynamics of bacterial isolates obtained from personal protective equipment (PPE), patient care environment (PCE) and general hospital sampling points (GHP).

Among the environmental samples (*n* = 139), the highest level of bacterial contamination was found on the outer surface of the physicians’ PPE suit (39.2%), nurses’ PPE suit (25.4%) and orderlies’ PPE suit (29.5%), as well as on handrails and levers of ICU beds (14.8%) and the surface of medical manipulation tables (11.2%). The temporal dynamics of contamination showed peaks at 10:00 and 18:00, coinciding with the periods of most intensive ICU work. (Figure 5).

### 3.3. SNP Analysis and Assessment of Recent Transmission

To confirm or exclude recent transmission of microorganisms between patients and the hospital environment, we used core-genome SNP distance analysis. Species with isolates present both on healthcare staff’s gloves and from patients that showed similar genetic profiles in preliminary typing—*K. pneumoniae* and *A. baumannii*—were selected for in-depth SNP analysis.

#### 3.3.1. Analysis of *Acinetobacter baumannii*

**Figure 6:**
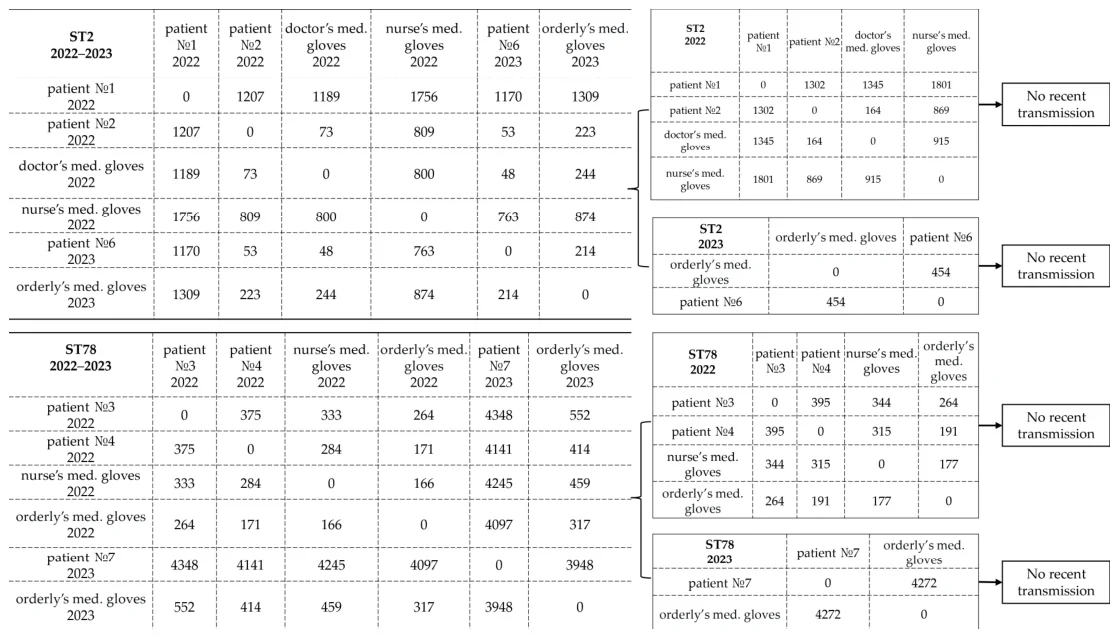
Pairwise SNP distances between *A. baumannii* isolates of STs: ST2 and ST78 obtained from patients and medical PPE gloves in 2022–2023.

For *A. baumannii*, pairs of isolates from “patient–healthcare staff hands” were analysed within the two most prevalent sequence types (ST2 and ST78) over both study phases. When separated by year (2022 and 2023), no reliable close relatedness was observed between patient isolates and isolates from healthcare staff hands (distances exceeded threshold values). This indicates that independent subpopulations of *A. baumannii* circulate in the hospital, despite the high similarity of resistance and virulence profiles among some isolates (Figure 6).

#### 3.3.2. Analysis of *Klebsiella pneumoniae*

**Figure 7:**
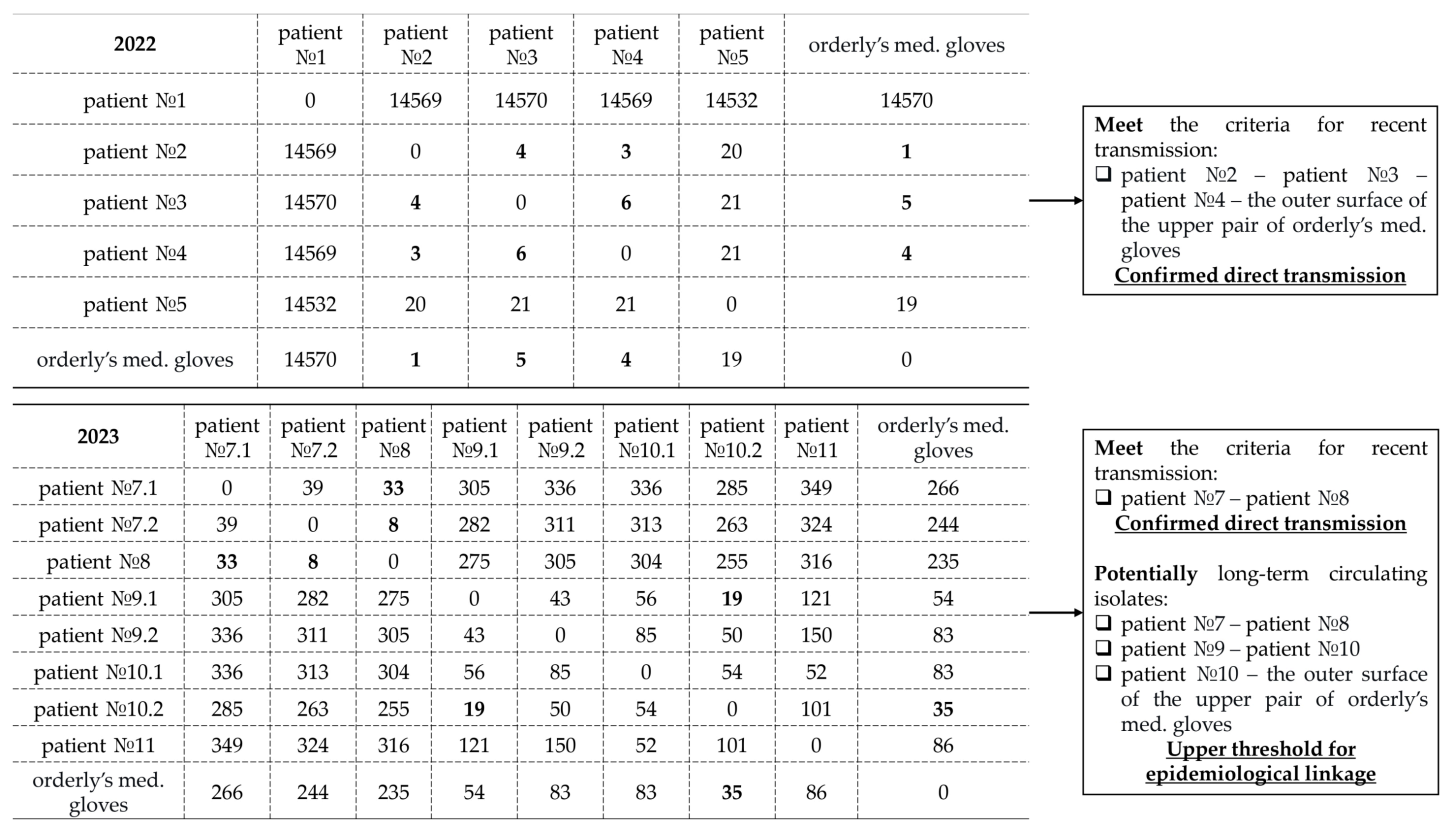
Pairwise SNP distances between *K. pneumoniae* isolates recovered from patients and medical PPE gloves in 2022–2023.

For *K. pneumoniae* in 2022, a high degree of genomic relatedness was found between isolates from three patients and from the orderly’s gloves (distance 1–6 SNPs), confirming recently transmission. In 2023, cases of recent transmission between two patients were also noted, as well as possible circulation of a *K. pneumoniae* subpopulation between patients and staff PPE (distances within the upper bound of relatedness). Thus, for *K. pneumoniae*, not only recently transmission from staff to patients was proven, but also circulation of isolates between patients and healthcare workers’ PPE (with possible subsequent transmission to other patients) (Figure 7).

### 3.4. Plasmid Pool and Its Role in the Spread of AMR

Plasmids play a key role in the horizontal transfer of AMR and virulence genes, enabling the rapid adaptation of bacterial populations to selective pressure in the hospital environment. In this study, an in silico analysis of plasmid replicons was performed for all isolates that underwent WGS.

All ten *E. coli* isolates (100%) carried plasmid replicons. The dominant types were IncFIA (60.0%), IncFIB (70.0%) and ColBS512 (50.0%). IncB/O/K/Z (20.0%), IncFII(pRSB107) (10.0%), IncHI2 (10.0%), and others were also detected. IncF group plasmids are widespread among *Enterobacteriaceae* and are often associated with the transfer of ESBL genes, including *bla*_CTX-M-15_, as well as carbapenemase genes [34,35]. In our study, isolates carrying IncFIA and IncFIB simultaneously harboured *bla*_CTX-M-15_ or *bla*_TEM-1_, confirming the role of these plasmids in the spread of ESBL phenotypes. The presence of ColBS512 (a replicon characteristic of colicinogenic plasmids) in 50.0% of isolates may indicate additional mechanisms of bacterial competition in the hospital setting [34]. The detection of IncB/O/K/Z—a group of plasmids often carrying heavy metal and aminoglycoside resistance genes—points to possible co-selection under the action of disinfectants [36].

Plasmid replicons were found in 17 of *S. aureus* isolates (85%). The most frequent were rep16 (55%), rep5a (40%), and rep20 (25%), rep21 (20%), repUS5 (20%), rep7c (15%) were also detected. The rep5a and rep16 plasmids are important vectors for the horizontal transfer of resistance genes, as confirmed by recent genomic studies [37]. rep20 and rep21, characteristic of the pT181 plasmid family, were associated with the *tet(38)* gene, conferring tetracycline resistance [38]. The rep7c replicon (pC194-like plasmid family) was linked to *fosB* and *murA*(G257D), confirming its role in the spread of fosfomycin resistance among staphylococci [39]. In isolates carrying repUS5, enterotoxin genes (*seg*, *sei*, *sen*, *seo*, *seu*) were additionally detected, suggesting a potential link between plasmids and virulence, although such an association is less common for staphylococci than for Gram-negative bacteria [40].

A wide diversity of plasmid replicons was found among *K. pneumoniae* isolates, reflecting the complex resistance structure of the population. The most common replicons were IncFIB(K) (71.7%), IncFII(K) (67.9%) and IncRNAI (64.2%). IncF family plasmids are major vectors for the spread of carbapenemases, including *bla*_KPC-3_, in *Enterobacteriaceae*, which is consistent with the high prevalence of IncFIB(K) and IncFII(K) among isolates of the dominant ST512 clone [41,42,43]. The IncX3 replicon, detected in 45.3% of isolates, is often associated with the transfer of *bla*_KPC-2_ and *bla*_NDM_ in various geographical regions [44]. The presence of IncHI1B (34.0%) is of interest because plasmids of this group often carry heavy metal and biocide resistance genes, including *qacEdelta1* [45]. IncL/M(pOXA-48) replicons, found in 13.2% of isolates, are classic carriers of *bla*_OXA-48_ [46]. Col440II (32.1%) and ColRNAI (64.2%) replicons belong to colicinogenic and low-copy-number plasmids that may participate in horizontal gene transfer, although their contribution to the spread of clinically relevant resistance is generally less pronounced [34].

Plasmid replicons were detected in 70% of *E. faecalis* isolates. The most frequent were rep9b (45%), repUS43 (35%). Replicons rep9a (25%), rep7a (25%) and rep6 (20%) were also detected. Family rep9 replicons are often involved in horizontal transfer of resistance genes among enterococci [47]. The absence of plasmids in 30% of isolates may indicate a chromosomal location of pathogenicity determinants.

All *P. mirabilis* isolates carried the IncC replicon, which has a broad host range and is often associated with the transfer of multidrug resistance genes, including *bla*_CTX-M_, *rmtB* and *sul* [48].

No plasmid replicons were detected in any of the *P. aeruginosa* isolates. This is typical for this species, as most resistance genes in *P. aeruginosa* are located on chromosomal integrated elements or on specific plasmids not detectable by standard replicon typing sets [31].

## 4. Discussion

The COVID-19 pandemic led to an unprecedented increase in HAIs and AMR, particularly in ICUs. Our study represents one of the first comprehensive analyses of the genetic landscape of OPs circulating in the ICUs of infectious diseases hospitals treating COVID-19 patients, using WGS. The data obtained allowed us not only to characterise the structure of the microbial population but also to identify key reservoirs, transmission pathway, and molecular mechanisms of pathogen adaptation within a closed hospital ecosystem.

### 4.1. Dominance of MDR Clones of Leading OPs

Among the obtained isolates OPs, *K. pneumoniae* and *A. baumannii* absolutely predominated (together accounting for 64.3% of isolates), which is consistent with global data on the leading role of these pathogens in the aetiology of HAIs during the pandemic [2,4]. Among *K. pneumoniae*, the dominant clone was ST512 (56.8% of typed isolates), characterised by the presence of the KPC-3 carbapenemase, a mutation in *pmrB*(R256G) associated with colistin resistance, and determinants of fluoroquinolone resistance (*gyrA*(S83I), *oqxAB*) and fosfomycin resistance (*fosA*). ST512 is one of the most successful global clones of KPC-producing *K. pneumoniae*, widespread in Europe, Asia and North America [41,42]. In our study, intra-hospital circulation of ST512 was established: isolates were recovered from nine patients, from seven PPE samples and from nine PCE, indicating insufficient effectiveness of infection control measures. Phylogenetic analysis revealed that the ST512 cluster was divided into two major subclusters. One subcluster comprised exclusively isolates from the third sampling phase, while the other contained isolates from all three phases. Notably, within the latter, the first-phase isolates originated from one hospital, while the related second-phase isolates came from the second hospital. Considering the bootstrap support (100 within each of the two groups and 88 between them), it can be hypothesised that these isolates share a common ancestor, which may indicate either inter-hospital transmission of the isolates or a common source of introduction. The risk of developing resistance to disinfectants is a serious concern. The detection of the *qacEdelta1* gene in the vast majority of isolates of the dominant ST512 clone (96%) is an important signal [49] that, nevertheless, requires careful interpretation.

The presence of the *qacE* gene or its variant *qacEdelta1* alone does not always mean that the bacterium is phenotypically resistant to quaternary ammonium compound (QAC)-based disinfectants. Nevertheless, its high prevalence in the hospital microbiota is an alarming marker, indicating potential selection and the possibility of resistance gene spread among other microorganisms. When such markers are detected, monitoring the resistance of epidemiologically significant strains to the disinfectants in use is necessary—genetic data must be complemented by direct phenotypic studies. As recommended by the Russian national regulations (SanPiN 3.3686-21, clause 3577), such findings should trigger an assessment of the need for disinfectant rotation, i.e., sequential replacement of the active substance from one chemical group with another, following susceptibility testing of the hospital strain to the newly selected agent. Accordingly, it is necessary to determine the minimum inhibitory concentrations of QACs for the isolates and to assess their survival under the working concentrations and exposure times of disinfectants as regulated by the national normative documents.

Among *A. baumannii*, the dominant STs were ST2 (35.7%) and ST78 (30.4%). ST2 is a “global” clone responsible for most hospital outbreaks of CRAB worldwide [50,51]. In our study, all ST2 isolates carried the OXA-23 carbapenemase, which is consistent with data on the wide spread of *bla*_OXA-23_ among ST2 in Europe, Asia and the Americas [52]. Interestingly, the second most frequent type, ST78, was characterised by the absence of *bla*_OXA-23_ but the presence of *bla*_OXA-72_ and *bla*_OXA-90_. ST78 is also known as a successful hospital clone, often associated with outbreaks in European countries [53], and its detection in our hospitals confirms the global spread of this clone.

For *E. coli*, dominance of clonal complex CC14 (83.3% of typed isolates) was revealed, including the potentially highly virulent and MDR clone ST1193. ST1193 is a recently described globally emerging clone belonging to phylogroup B2, often associated with community-acquired and hospital-acquired urinary tract infections and bacteraemia [54,55]. In our study, ST1193 isolates carried *bla*_CTX-M-15_ (ESBL) and mutations in *parC*(80I) and conferring fluoroquinolone resistance, which is characteristic of this clone [56].

*Staphylococcus aureus* in our study was represented by several CCs: CC15 (30%), CC8 (15%), CC22 (10%) and CC97 (10%). MRSA were detected exclusively among CC22 and some non-defined isolates. CC22 (specifically its variant EMRSA-15) is one of the most widespread hospital MRSA clones in Europe and worldwide [57]. During the COVID-19 pandemic, outbreaks of MRSA CC22 in COVID-19 units have been reported, linked to breaches in infection control protocols [58]. CC15 and CC8 (the latter includes the well-known USA300 clone) are more often associated with community-acquired infections; however, their detection on staff PPE indicates a potential role in hospital transmission [59]. Interestingly, CC97, traditionally associated with bovine mastitis, was isolated both from a patient and from PPE, which may indicate possible zoonotic transfer or adaptation of this clone to humans [60].

*Enterococcus faecalis* was characterised by a polyclonal structure with predominance of ST30 (25% of typed isolates) and ST40 (15%), which is consistent with a global meta-analysis showing a wide distribution of various STs of this species in clinical and ecological niches [61]. The high frequency of *lsa(A)* (85%) and *erm(B)* (45%) genes reflects the selective pressure typical of ICUs, where lincosamides and macrolides are widely used. The detection of the cytolysin gene (*cyl*) in 45% of isolates has clinical significance, as cytolysin is associated with increased virulence and mortality in enterococcal infections [62].

Both isolates of *E. faecium* belonged to ST52—a hospital clone previously described as associated with nosocomial infections in Europe [63]. In our study, the ST52 isolates were identical in their resistance profile and plasmid pool, suggesting a clonal origin. The absence of virulence genes is typical for *E. faecium*, where pathogenicity is more linked to antibiotic resistance and persistence capacity than to toxigenicity [64]. The detection of ST52 on staff PPE indicates a potential role of this clone in the development of hospital infections.

Among *P. mirabilis*, ST269 and ST234 were typed—clones previously described in hospitals in Asia and Europe [65]. All isolates carried ESBL genes (*bla*_CTX-M-3_, *bla*_CTX-M-14_, *bla*_TEM-1_), which is consistent with the global trend of increasing ESBL-producing *P. mirabilis* strains, especially in Asian countries where the prevalence of *bla*_CTX-M-14_ reaches 11.3% [49,66]. The presence of *rmtB* (16S rRNA methyltransferase) in ST269 isolates is an alarming marker, as this gene confers high-level resistance to all aminoglycosides and has previously been detected in *P. mirabilis* in Taiwan and Korea [49,66]. The detection of IncC plasmids in all isolates confirms their key role in the spread of multidrug resistance in this species [65].

*Pseudomonas aeruginosa* was represented by international high-risk clones: ST235, ST654 and ST549. ST235 is the dominant high-risk clone worldwide, associated with the production of various carbapenemases and high virulence (production of exotoxin *ExoU*) [67,68]. ST654 is also among the top ten high-risk clones and is often associated with *bla*_VIM-2_ metallo-β-lactamase [67,69]. In our study, the ST235 isolate carried *bla*_GES-1_, while ST654 isolates carried *bla*_VIM-2_, confirming the circulation of different carbapenemases in the same unit. The absence of plasmid replicons in all *P. aeruginosa* isolates is not unexpected: most resistance genes in this species are located on chromosomal integrated elements or on specific plasmids that are not detectable by standard replicon typing sets [70].

### 4.2. Personal Protective Equipment and the Hospital Environment as Critical Reservoirs of Pathogens

One of the most significant findings of our study is the high level of contamination of healthcare staff’s PPE: 68.4% of environmental isolates were obtained from the surfaces of PPE suits and medical gloves. Particularly frequent isolation from PPE was observed for *A. baumannii* (47–64% of ST2 and ST78 isolates were found on PPE) and *S. aureus* (60–100% of CC8, CC22, CC15 and non-defined isolates were found on PPE). These data are consistent with a systematic review showing that PPE contamination levels can reach 77.5% and that prolonged PPE use (up to 12 h or more) during the pandemic significantly increases the risk of pathogen transmission [8].

Phylogenetic and SNP analyses confirmed direct epidemiological links between isolates obtained from PPE and those from patients. Specifically, for *K. pneumoniae*, cases with core genome differences of 1–6 SNPs were identified, providing strong evidence of recent transmission [31]. These data highlight that PPE acts not only as a passive barrier but also as an active reservoir and vector for nosocomial transmission of pathogens, necessitating enhanced control over their use and replacement [71].

The temporal dynamics of PPE contamination showed peaks at 10:00 and 18:00, coinciding with the periods of most intensive ICU work (shift changes, invasive procedures, ward rounds). Similar patterns have been described in studies of surface contamination in ICUs [72]. The high level of contamination in the evening (18:00, 22:00) may reflect a cumulative effect of microorganism build-up during the work shift and calls for a review of PPE change frequency and disinfection procedures.

### 4.3. Value of Whole-Genome Sequencing and Core Genome SNP Analysis for Epidemiological Surveillance

Our study demonstrated the advantages of WGS over traditional typing methods. Classical MLST allowed only a rough stratification of isolates but did not enable assessment of microevolutionary changes. SNP analysis revealed significant heterogeneity within *A. baumannii* ST2 (>100 allele differences), indicating long-term circulation and adaptation of the clone within the hospital. Similar heterogeneity has been described for other hospital clones of *A. baumannii* [73].

For *K. pneumoniae*, SNP analysis not only confirmed recent transmission (differences of 1–6 SNPs) but also revealed cases with 35–349 SNP differences, which may correspond to the circulation of genetically related but not identical lineages within a single outbreak or reflect long-term persistence in the ICU [74]. In the literature, a threshold of 10–15 SNPs is commonly used to differentiate recent transmission from background circulation, although different criteria may apply for different species and time scales [31,75].

The application of WGS also allowed a unified characterisation of the resistome, virulome and plasmid pool of all isolates, providing an opportunity to assess the potential for horizontal gene transfer. The high frequency of IncF group plasmid replicons in *E. coli* and *K. pneumoniae*, as well as rep5a and rep16 in *S. aureus*, confirms their key role in the spread of resistance genes in the hospital environment [42]. The detection of biocide resistance determinants (*qacEdelta1* in *K. pneumoniae*) further explains the ability of these isolates to persist for long periods on environmental surfaces [49].

### 4.4. Comparison with Results of Other Studies During the Pandemic

Our data are consistent with the results of multicentre studies showing an increased frequency of MDR OPs isolation during the COVID-19 pandemic. A study conducted in Italy involving 1662 patients reported increased resistance of *A. baumannii* to gentamicin (from 70.9% to 91.3%) and colistin (from 3.2% to 12.3%), as well as increased ICU mortality [2]. Another study covering 41 Brazilian hospitals also showed dominance of *A. baumannii* and *K. pneumoniae* on ICU surfaces, with the authors highlighting the role of PPE as a major reservoir [76].

The spread of the *A. baumannii* ST2 clone carrying *bla*_OXA-23_ during the pandemic has been confirmed in studies from Croatia, Switzerland and Mexico [77,78,79], indicating the global nature of this phenomenon. The detection of *A. baumannii* ST78 with *bla*_OXA-72_ is also consistent with data on the spread of this clone in Europe [53].

Regarding *E. coli* ST1193, its high proportion among isolates in our study (40% of typed isolates) is comparable to data from China and the USA, where ST1193 has become the second most frequent ESBL-producing clone after ST131 [55,56]. Interestingly, in our study ST1193 formed a separate phylogenetic cluster, which may indicate endemic circulation of this lineage in the hospital.

For *S. aureus*, the dominance of CC22 MRSA and CC15 among isolates from PPE agrees with the results of a recent study from China, where CC22 was the most frequent MRSA clone in COVID-19 units, while CC15 predominated among methicillin-susceptible isolates [59]. The detection of CC97 in our hospital requires further investigation, as this clone is traditionally associated with livestock, but cases of human isolation have been described in recent years [61].

### 4.5. Study Limitations

Our study has several limitations. First, the study was conducted in two infectious diseases hospitals located in different cities of the Sverdlovsk region, which may limit the generalisability of the results to other regions and types of hospitals. Second, resistance determination was performed in silico based on genomic data, without phenotypic confirmation by disc diffusion or serial dilution methods. However, ResFinder and AMRFinderPlus have high predictive value (sensitivity and specificity >95% for most antibiotic classes) [21,22]. Third, the time frame of the study (2021–2023) limits the ability to assess long-term trends, especially in the post-pandemic period. Fourth, single isolates of some species (*K. variicola*) do not allow general conclusions to be drawn about their role in the epidemic process. Moreover, for seven out of 175 isolates (4%), complete genome assemblies could not be obtained due to short contigs that did not cover the full genome; consequently, their sequences were not deposited in GenBank, which did not affect the main conclusions of the study.

## 5. Conclusions

Thus, our study provided a comprehensive genetic characterisation of OPs circulating in the ICUs of COVID-19 hospitals, revealed the dominance of MDR clones of international significance, confirmed the key role of PPE and the hospital environment in infection transmission, and demonstrated the high resolution of WGS and SNP analysis for epidemiological surveillance. The obtained results justify the need to strengthen infection control measures and to implement genomic monitoring to prevent the spread of MDR OPs in infectious disease hospitals.

## Figures and Tables

**Table 1:** Summary of key genetic characteristics of the main opportunistic pathogen species.

Species	Dominant Clones (ST/CC)	Key Resistance Determinants	Key Virulence Factors	Dominant Plasmid Replicons
*A. baumannii*	ST2 (40%), ST78 (28.3%), ST19 (3.3%)	*bla*OXA-23 (ST2), *bla*OXA-72/90 (ST78), *armA*	*csu*, bas genes, T6SS, *ompA*, *pmrB*, *ade*, *abe*	None detected
*K. pneumoniae*	ST512 (47.2%), ST15, ST101, ST307, ST395, ST1190	*bla*KPC-3, *pmrB*(R256G), *gyrA*(S83I), *oqxAB*, *qacEdelta1*, *fosA* (ST512); ESBL in others	*ecp*, *ompA*; *fyuA*, *ybt*, *irp*, *ent*, *fepC* in ST512; no toxins	IncFIB(K), IncFII(K), IncRNAI
*S. aureus*	CC15 (30%), CC8 (15%), CC22 (10%), CC97 (10%)	*mecA*, *blaZ*, *tet*(38), *fosB*	*aur* (90%), *hlgABC* (90%), enterotoxins (10–30%)	rep16, rep5a
*E. faecalis*	ST30 (25%), ST40 (15%), multiple STs	*lsa(A)* (85%), *dfrE* (80%), *tetM* (60%), *erm(B)* (45%)	*ebpABC*, *fsrABC*, *cyl* (45%), *gelE*, *sprE*	rep9b, repUS43
*E. coli*	CC14 (incl. ST1193)	*bla*CTX-M-15, *marR*(S3N), fluoroquinolone mutations	*fimH* (100%), *yehABCD*, *traT* (78%)	IncFIA, IncFIB, ColBS512
*P. mirabilis*	ST269 (60%), ST234 (40%)	*bla*CTX-M-3/14, *bla*TEM-1, *rmtB*	*mrpA*, *pmfA*, *fimH*, T6SS, urease	IncC
*P. aeruginosa*	ST654 (50%), ST235 (25%), ST549 (25%)	*bla*VIM-2 (ST654), *bla*GES-1 (ST235)	Extensive: T3SS, T6SS, *toxA*, *exoT*/*S*/*Y*	None detected
*E. faecium*	ST52	*aac(6′)-I*, *msr*(*C*)	None detected	repUS15, rep1, rep14b
*K. variicola*	ST4101 (single isolate)	*bla*LEN-2, *oqxAB*, *fosA*	*ecp* operon, *ompA*; *fyuA*, *ybt*, *irp*, ent, *fepC*	IncFIB(K), IncFII(K), Col(pHAD28)

## Data Availability

The raw sequencing data generated in this study have been deposited in the NCBI SRA database under BioProject accession number PRJNA1165946 (*A. baumannii*), PRJNA1180224 (*E. coli*), PRJNA1178453 (*S. aureus*), PRJNA1348999 (*K. pneumoniae* and *K. variicola*), PRJNA1348956 (*E. faecalis*), PRJNA1348989 (*E. faecium*), PRJNA1348996 (*P. mirabilis*), PRJNA1348997 (*P. aeruginosa*).

## Supplementary Materials

The following supporting information can be downloaded at: https://www.mdpi.com/article/10.3390/pathogens15080845/s1, Table S1: Complete list of antimicrobial resistance genes for all 175 isolates. Table S2: Complete list of virulence determinants for all 175 isolates. Table S3: Complete list of plasmid replicons for all 175 isolates.

## Abbreviations

The following abbreviations are used in this manuscript:

AMR	Antimicrobial resistance
CC	Clonal complex
CRAB	Carbapenem-resistant *A. baumannii*
cgMLST	Core-genome MLST
ICUs	Intensive care units
HAIs	Healthcare-associated infections
OP	Opportunistic pathogen
MDR	Multidrug-resistant
MLST	Multilocus sequence typing
PPE	Personal protective equipment
SNP	Single-nucleotide polymorphism
ST	Sequence type
WGS	Whole-genome sequencing
PCE	Patient care environment
GHP	General hospital point

## Appendix A

**Table A1:** Sampling points.

ID	Stage	Selection Group	Selection Point	GB Accession
ab_01	III	Patient	sputum	JBITPY000000000
ab_02	III	Patient	sputum	JBITPX000000000
ab_03	II	Patient	urethra	JBITPW000000000
ab_04	II	Patient	sputum	JBHYBP000000000
ab_05	II	Patient	sputum	JBHYBQ000000000
ab_06	II	Patient	sputum	JBHYBR000000000
ab_07	II	Patient	oropharyngeal swab	JBITPZ000000000
ab_08	II	Patient	oropharyngeal swab	JBHYBS000000000
ab_09	II	Patient	oropharyngeal swab	JBHYBT000000000
ab_10	III	PCE	the outer surface or the ventilator	JBRYPU000000000
ab_11	III	PCE	the surface of medical manipulation table	JBITQH000000000
ab_12	III	PPE	the outer surface or the orderly’s PPE suit	JBITQG000000000
ab_13	III	PPE	the outer surface or the orderly’s PPE suit	JBITQA000000000
ab_14	III	PPE	the outer surface or the upper pair of orderly’s medical gloves	JBHYBU000000000
ab_15	III	PCE	handrails and adjustment levers on an ICU bed	JBHYBX000000000
ab_16	III	PPE	the outer surface or the nurse’s PPE suit	JBITQK000000000
ab_17	III	PPE	the outer surface or the doctor’s PPE suit	JBHYBW000000000
ab_18	III	PCE	handrails and adjustment levers on an ICU bed	JBHYCH000000000
ab_19	III	PCE	handrails and adjustment levers on an ICU bed	JBHYCE000000000
ab_20	III	PCE	the outer surface or the ventilator	JBHYBY000000000
ab_21	III	PCE	the surface of medical manipulation table	JBITQZ000000000
ab_22	III	PPE	the outer surface or the orderly’s PPE suit	JBITQX000000000
ab_23	III	PPE	the outer surface or the orderly’s PPE suit	JBITQW000000000
ab_24	III	PPE	the outer surface or the upper pair of orderly’s medical gloves	JBITQV000000000
ab_25	III	PPE	the outer surface or the nurse’s PPE suit	JBHYCJ000000000
ab_26	III	PPE	the outer surface or the nurse’s PPE suit	JBITRA000000000
ab_27	II	PCE	the surface of medical manipulation table	JBITQB000000000
ab_28	II	PCE	the outer surface of the medical syringe dispenser	JBITQC000000000
ab_29	II	PPE	the outer surface or the upper pair of doctor’s medical gloves	JBHYBV000000000
ab_30	II	PPE	the outer surface or the upper pair of nurse’s medical gloves	JBITQD000000000
ab_31	II	PPE	the outer surface or the orderly’s PPE suit	JBITQE000000000
ab_32	II	PPE	the outer surface or the doctor’s PPE suit	JBITQF000000000
ab_33	II	PCE	handrails and adjustment levers on an ICU bed	JBITQI000000000
ab_34	II	PCE	the outer surface of the medical syringe dispenser	—
ab_35	II	GHP	ICU door handles	JBITQJ000000000
ab_36	II	PCE	the surface of medical manipulation table	JBITQL000000000
ab_37	II	PCE	handrails and adjustment levers on an ICU bed	—
ab_38	II	PCE	the outer surface or the ventilator	JBITQM000000000
ab_39	II	PCE	handrails and adjustment levers on an ICU bed	JBHYBZ000000000
ab_40	II	PCE	the outer surface or the ventilator	JBHYCA000000000
ab_41	II	PPE	the outer surface or the upper pair of orderly’s medical gloves	JBHYCB000000000
ab_42	II	PPE	the outer surface or the upper pair of nurse’s medical gloves	JBITQN000000000
ab_43	II	PPE	the outer surface or the nurse’s PPE suit	JBITQO000000000
ab_44	II	PPE	the outer surface or the doctor’s PPE suit	JBHYCC000000000
ab_45	II	PCE	handrails and adjustment levers on an ICU bed	JBHYCD000000000
ab_46	II	PCE	the outer surface or the ventilator	JBRYPS000000000
ab_47	II	PCE	the surface of medical manipulation table	JBITQP000000000
ab_48	II	PCE	handrails and adjustment levers on an ICU bed	JBITQQ000000000
ab_49	II	GHP	the outer surface of the suction unit	JBHYCF000000000
ab_50	II	GHP	dispensers for liquid soap and hand sanitizer	JBITQR000000000
ab_51	II	PPE	the outer surface or the nurse’s PPE suit	JBITQS000000000
ab_52	II	PPE	the outer surface or the orderly’s PPE suit	JBHYCG000000000
ab_53	III	PCE	handrails and adjustment levers on an ICU bed	JBRYPT000000000
ab_54	II	PPE	the outer surface or the doctor’s PPE suit	—
ab_55	II	PCE	handrails and adjustment levers on an ICU bed	JBITQT000000000
ab_56	II	GHP	ICU door handles	JBITQU000000000
ab_57	II	PPE	the outer surface or the upper pair of doctor’s medical gloves	JBHYCI000000000
ab_58	II	PCE	the outer surface or the ventilator	JBSHXN000000000
ab_59	II	PCE	the surface of medical manipulation table	JBITQY000000000
ab_60	II	PPE	the outer surface or the orderly’s PPE suit	JBHYCK000000000
kp_01	III	Patient	sputum	JBRXXF000000000
kp_02	III	Patient	sputum	JBRXXG000000000
kp_03	III	Patient	sputum	JBRXXD000000000
kp_04	III	Patient	sputum	JBRXXE000000000
kp_05	III	Patient	sputum	JBRXXB000000000
kp_06	III	Patient	sputum	JBRXWZ000000000
kp_07	III	Patient	sputum	JBRXXA000000000
kp_08	III	Patient	sputum	JBRXWW000000000
kp_09	III	Patient	sputum	JBRXWV000000000
kp_10	III	Patient	sputum	JBRXWX000000000
kp_11	II	Patient	sputum	JBRXXC000000000
kp_12	II	Patient	sputum	JBRXWY000000000
kp_13	II	Patient	sputum	JBRXWU000000000
kp_14	II	Patient	sputum	JBRXWT000000000
kp_15	II	Patient	sputum	JBRXWS000000000
kp_16	III	PCE	handrails and adjustment levers on an ICU bed	JBRYKD000000000
kp_17	III	PPE	the outer surface or the orderly’s PPE suit	JBRYKG000000000
kp_18	III	PPE	the outer surface or the upper pair of orderly’s medical gloves	JBRYKH000000000
kp_19	III	PCE	the outer surface or the ventilator	JBRYJY000000000
kp_20	III	PCE	the outer surface of the medical syringe dispenser	JBRYJZ000000000
kp_21	III	PCE	handrails and adjustment levers on an ICU bed	JBRYKA000000000
kp_22	III	PPE	the outer surface or the orderly’s PPE suit	JBRYKB000000000
kp_23	III	PPE	the outer surface or the nurse’s PPE suit	JBRYKC000000000
kp_24	III	PCE	handrails and adjustment levers on an ICU bed	JBSBGB000000000
kp_25	III	PCE	handrails and adjustment levers on an ICU bed	JBRYJR000000000
kp_26	III	PCE	handrails and adjustment levers on an ICU bed	JBRYJX000000000
kp_27	III	PPE	the outer surface or the nurse’s PPE suit	JBRYJL000000000
kp_28	III	PCE	the outer surface or the ventilator	JBRYJQ000000000
kp_29	II	PPE	the outer surface or the doctor’s PPE suit	JBRYKI000000000
kp_30	II	PCE	the outer surface of the medical syringe dispenser	JBRYKF000000000
kp_31	II	GHP	ICU electric light switches	JBRYKE000000000
kp_32	II	PCE	the outer surface of the medical syringe dispenser	JBRYJW000000000
kp_33	II	PPE	the outer surface or the nurse’s PPE suit	JBRYJV000000000
kp_34	II	PPE	the outer surface or the orderly’s PPE suit	JBRYJU000000000
kp_35	II	PPE	the outer surface or the doctor’s PPE suit	JBRYJT000000000
kp_36	II	PCE	the surface of medical manipulation table	JBRYJS000000000
kp_37	II	PCE	the outer surface or the ventilator	JBRYJP000000000
kp_38	II	GHP	the outer surface of the suction unit	JBRYJO000000000
kp_39	II	PCE	the outer surface or the ventilator	JBRYJN000000000
kp_40	II	PCE	handrails and adjustment levers on an ICU bed	JBRYJM000000000
kp_41	II	PCE	the outer surface or the ventilator	JBUAPN000000000
kp_42	II	GHP	the outer surface of the suction unit	JBRYJK000000000
kp_43	II	GHP	dispensers for liquid soap and hand sanitizer	JBRYJJ000000000
kp_44	II	PPE	the outer surface or the upper pair of orderly’s medical gloves	JBRYJI000000000
kp_45	II	PCE	handrails and adjustment levers on an ICU bed	JBRYJH000000000
kp_46	II	PCE	the outer surface or the ventilator	JBRYJG000000000
kp_47	I	GHP	clinical doctor’s work-space	JBRYJF000000000
kp_48	I	PPE	the outer surface or the doctor’s PPE suit	JBRYJE000000000
kp_49	I	PPE	the outer surface or the upper pair of doctor’s medical gloves	JBRYJD000000000
kp_50	I	GHP	the outer surface of the suction unit	JBRYJC000000000
kp_51	I	PPE	the outer surface or the doctor’s PPE suit	JBRYJB000000000
kp_52	I	PPE	the outer surface or the nurse’s PPE suit	JBRYJA000000000
kp_53	I	PPE	the outer surface or the orderly’s PPE suit	JBRYIZ000000000
sa_01	II	Patient	oropharyngeal swab	JBIYQB000000000
sa_02	II	Patient	sputum	JBIYQA000000000
sa_03	II	Patient	sputum	JBIYPZ000000000
sa_04	III	PCE	handrails and adjustment levers on an ICU bed	JBIYQL000000000
sa_05	III	PPE	the outer surface or the orderly’s PPE suit	JBIYQM000000000
sa_06	III	PPE	the outer surface or the orderly’s PPE suit	JBIYQN000000000
sa_07	III	PPE	the outer surface or the upper pair of nurse’s medical gloves	JBIYQO000000000
sa_08	III	PCE	the surface of medical manipulation table	JBIYQJ000000000
sa_09	III	PPE	the outer surface or the doctor’s PPE suit	JBIYQK000000000
sa_10	III	PCE	handrails and adjustment levers on an ICU bed	JBIYQQ000000000
sa_11	II	PPE	the outer surface or the nurse’s PPE suit	—
sa_12	II	PPE	the outer surface or the upper pair of orderly’s medical gloves	JBIYQP000000000
sa_13	II	PPE	the outer surface or the doctor’s PPE suit	JBIYRD000000000
sa_14	II	PPE	the outer surface or the upper pair of nurse’s medical gloves	JBIYQI000000000
sa_15	II	PPE	the outer surface or the doctor’s PPE suit	JBIYQH000000000
sa_16	II	PPE	the outer surface or the doctor’s PPE suit	JBIYQG000000000
sa_17	II	PPE	the outer surface or the doctor’s PPE suit	JBIYQF000000000
sa_18	I	PPE	the outer surface or the upper pair of doctor’s medical gloves	JBIYQE000000000
sa_19	I	PPE	the outer surface or the upper pair of nurse’s medical gloves	JBIYQD000000000
sa_20	I	PPE	the outer surface or the nurse’s PPE suit	JBIYQC000000000
ec_01	II	Patient	urine	JBRYYL000000000
ec_02	II	Patient	sputum	—
ec_03	II	PPE	the outer surface or the upper pair of doctor’s medical gloves	—
ec_04	I	PCE	the surface of medical manipulation table	JBIYQW000000000
ec_05	I	PPE	the outer surface or the doctor’s PPE suit	JBIYQV000000000
ec_06	I	PPE	the outer surface or the nurse’s PPE suit	JBRYYK000000000
ec_07	I	PCE	handrails and adjustment levers on an ICU bed	JBIYQT000000000
ec_08	I	PPE	the outer surface or the nurse’s PPE suit	JBIYQS000000000
ec_09	I	PCE	the surface of medical manipulation table	JBIYQR000000000
ec_10	I	PPE	the outer surface or the doctor’s PPE suit	—
efs_01	III	Patient	sputum	JBRXJJ000000000
efs_02	II	Patient	sputum	JBRXJK000000000
efs_03	III	PCE	handrails and adjustment levers on an ICU bed	JBUAPO000000000
efs_04	II	Patient	sputum	JBRXJI000000000
efs_05	I	PPE	the outer surface or the upper pair of doctor’s medical gloves	JBRXKA000000000
efs_06	I	PPE	the outer surface or the upper pair of nurse’s medical gloves	JBRXJZ000000000
efs_07	I	PPE	the outer surface or the doctor’s PPE suit	JBRXJY000000000
efs_08	I	PPE	the outer surface or the orderly’s PPE suit	JBRXJX000000000
efs_09	I	PCE	the surface of medical manipulation table	JBRXJW000000000
efs_10	I	PPE	the outer surface or the doctor’s PPE suit	JBRXJV000000000
efs_11	I	PPE	the outer surface or the upper pair of doctor’s medical gloves	JBRXJU000000000
efs_12	I	PPE	the outer surface or the doctor’s PPE suit	JBRXJT000000000
efs_13	I	PPE	the outer surface or the nurse’s PPE suit	JBRXJS000000000
efs_14	I	PPE	the outer surface or the upper pair of doctor’s medical gloves	JBRXJR000000000
efs_15	I	PPE	the outer surface or the doctor’s PPE suit	JBRXJQ000000000
efs_16	I	PPE	the outer surface or the orderly’s PPE suit	JBRXJP000000000
efs_17	I	PCE	the surface of medical manipulation table	JBRXJO000000000
efs_18	I	PCE	handrails and adjustment levers on an ICU bed	JBRXJN000000000
efs_19	I	PPE	the outer surface or the doctor’s PPE suit	JBRXJM000000000
efs_20	I	PPE	the outer surface or the nurse’s PPE suit	JBRXJL000000000
efm_01	I	PPE	the outer surface or the upper pair of doctor’s medical gloves	JBRXVA000000000
efm_02	I	PPE	the outer surface or the upper pair of nurse’s medical gloves	JBRXUZ000000000
pm_01	II	Patient	sputum	JBWGWX000000000
pm_02	II	Patient	sputum	JBWGWW000000000
pm_03	II	PPE	the outer surface or the nurse’s PPE suit	JBWGXA000000000
pm_04	II	PCE	the surface of medical manipulation table	JBWGWZ000000000
pm_05	II	PPE	the outer surface or the upper pair of orderly’s medical gloves	JBWGWY000000000
pa_01	II	Patient	sputum	JBRXVJ000000000
pa_02	II	Patient	sputum	JBRXVI000000000
pa_03	II	GHP	oxygen pipeline valves	JBRXVH000000000
pa_04	I	PPE	the outer surface or the doctor’s PPE suit	JBRXVG000000000
kv_01	II	PPE	the outer surface or the upper pair of doctor’s medical gloves	JBSCEI000000000

Notes: ab—*A. baumannii*, kp—*K. pneumoniae*, sa –*S. aureus*, ec—*E. coli*, efs—*E. faecalis*, efm—*E. faecium*, pm—*P. mirabilis*, pa—*P. aeruginosa*, kv—*K. variivola*; PPE—personal protective equipment; PCE—patient care environment; GHP—general hospital point. Stage refers to the sampling phase: I—first phase (2021), II—second phase (2022), III—third phase (2023).

## References

[1] Peconi C., Martini E., Sarti D., Prospero E. (2025). Impact of the COVID-19 pandemic on healthcare-associated infections and multidrug-resistant microorganisms in Italy: A systematic review. J. Infect. Public Health.

[2] Çapar A., Özyiğitoğlu D., Başlılar Ş. (2025). Investigation of antibiotic resistance against pathogens isolated from respiratory samples in intensive care units after SARS-CoV-2 pandemic. BMC Infect. Dis..

[3] Kutyrev V.V., Popova A.Y., Smolensky V.Y., Ezhlova E.B., Demina Y.V., Safronov V.A., Karnaukhov I.G., Ivanova A.V., Shcherbakova S.A. (2020). Epidemiological Peculiarities of New Coronavirus Infection (COVID-19). Communication 2: Peculiarities of epidemic process development in conjunction with performed anti-epidemic measures around the world and in the Russian Federation. Probl. Osob. Opasnykh Infektsii.

[4] Lansbury L., Lim B., Baskaran V., Lim W.S. (2020). Co-infections in people with COVID-19: A systematic review and meta-analysis. J. Infect..

[5] Farzana R., Harbarth S.J., Yu L.-M., Carretto E., Moore E.C., Feasey N.A., Gales A.C., Galal U., Ergonul O., Yong D. (2025). The impact of the COVID-19 pandemic on antimicrobial usage: An international patient-level cohort study. JAC Antimicrob. Resist..

[6] Abubakar U., Al-Anazi M., Alanazi Z., Rodríguez-Baño J. (2023). Impact of COVID-19 pandemic on multidrug resistant gram positive and gram negative pathogens: A systematic review. J. Infect. Public Health.

[7] Emdin F., Orubu E.S.F., Van Katwyk S.R., Strong K., Shaver N., Abdullah K., Asamoah G., Skidmore B., Chan E., Poirier M.J.P. (2025). COVID-19 impact on AMR: A rapid scoping review, equity analysis and evidence gap map study. BMJ Glob. Health.

[8] Orsel L.M., Severin J.A., Knoester M., Lokate M., Voss A., Haanappel C.P., van Kampen J.J.A., Haagmans B.L., Koopmans M.P.G., Veldkamp K.E. (2025). The role of gowns in preventing nosocomial transmission of respiratory viruses: A systematic review. J. Hosp. Infect..

[9] Dhandapani S., Priyadarshi K., Benedict Vinothini A., Rajshekar D., Punnen S.A., Sastry A.S. (2024). Donning of personal protective equipment under direct supervision—A quality improvement study in COVID-ICUs of a tertiary care facility, South India. IP Int. J. Med. Microbiol. Trop. Dis..

[10] Shropshire W.C., Hanson B.M., Shelburne S.A. (2025). Genome-wide approaches to bacterial strain typing: A history and review of recent methodological advances. Curr. Opin. Infect. Dis..

[11] Szarvas J., Bartels M.D., Westh H., Lund O. (2021). Rapid Open-Source SNP-Based Clustering Offers an Alternative to Core Genome MLST for Outbreak Tracing in a Hospital Setting. Front. Microbiol..

[12] Kaplan E., Lurie-Weinberger M.N., Kastel O., Cohen A., Schwartz D., Keren-Paz A., Carmeli Y. (2025). Enterobacter cloacae complex taxonomic incongruence may lead to misidentification of outbreaks and hinder infection control in real time. Eur. J. Clin. Microbiol. Infect. Dis..

[13] Waggle K.D., Griffith M.P., Rokes A.B., Sundermann A., Cooper V., Harrison L., Pless L. (2025). Methods for cost-efficient, whole genome sequencing surveillance for enhanced detection of outbreaks in a hospital setting. medRxiv.

[14] Mustapha M.M., Srinivasa V.R., Griffith M.P., Cho S.-T., Evans D.R., Waggle K., Ezeonwuka C., Snyder D.J., Marsh J.W., Harrison L.H. (2022). Genomic Diversity of Hospital-Acquired Infections Revealed Through Prospective Whole-Genome Sequencing-Based Surveillance. mSystems.

[15] Smirnova S.S., Zhuikov N.N., Egorov I.A., Pushkareva N.A., Semenov A.V. (2022). Sampling Scheme of Flushes from Environmental Objects for Simultaneous Assessment of Viral and Bacterial Contamination: No. 2022501675. Russian Federation Industrial Design Patent.

[16] Smirnova S.S., Avdyunin D.D., Holmanskikh M.V., Stagilskaya Y.S., Zhuikov N.N., Itani T.M. (2025). Genetic Characteristics of *Acinetobacter baumannii* Isolates Circulating in an Intensive Care Unit of an Infectious Diseases Hospital During the COVID-19 Pandemic. Pathogens.

[17] (2011). Methods for Sanitary-Bacteriological Studies of Environmental Objects, Air, and Sterility Control in Healthcare Institutions.

[18] Vasilinetc I., Prjibelski A.D., Gurevich A., Korobeynikov A., Pevzner P.A. (2015). Assembling short reads from jumping libraries with large insert sizes. Bioinformatics.

[19] Astashyn A., Tvedte E.S., Sweeney D., Sapojnikov V., Bouk N., Joukov V., Mozes E., Strope P.K., Sylla P.M., Wagner L. (2024). Rapid and Sensitive Detection of Genome Contamination at Scale with FCS-GX. Genome Biol..

[20] Chen S., Zhou Y., Chen Y., Gu J. (2018). fastp: An ultra-fast all-in-one FASTQ preprocessor. Bioinformatics.

[21] Bortolaia V., Kaas R.S., Ruppe E., Roberts M.C., Schwarz S., Cattoir V., Philippon A., Allesoe R.L., Rebelo A.R., Florensa A.F. (2020). ResFinder 4.0 for predictions of phenotypes from genotypes. J. Antimicrob. Chemother..

[22] Feldgarden M., Brover V., Gonzalez-Escalona N., Frye J.G., Haendiges J., Haft D.H., Hoffmann M., Pettengill J.B., Prasad A.B., Tillman G.E. (2021). AMRFinderPlus and the Reference Gene Catalog facilitate examination of the genomic links among antimicrobial resistance, stress response, and virulence. Sci. Rep..

[23] Seemann T. (2016). ABRicate: Mass Screening of Contigs for Antibiotic Resistance Genes. GitHub. https://github.com/tseemann/abricate.

[24] Carattoli A., Hasman H. (2020). PlasmidFinder and in Silico pMLST: Identification and Typing of Plasmid Replicons in Whole-Genome Sequencing (WGS). Methods Mol. Biol..

[25] Seemann T. (2014). Prokka: Rapid prokaryotic genome annotation. Bioinformatics.

[26] Page A.J., Cummins C.A., Hunt M., Wong V.K., Reuter S., Holden M.T.G., Fookes M., Falush D., Keane J.A., Parkhill J. (2015). Roary: Rapid large-scale prokaryote pan genome analysis. Bioinformatics.

[27] Stamatakis A. (2014). RAxML version 8: A tool for phylogenetic analysis and post-analysis of large phylogenies. Bioinformatics.

[28] Kalyaanamoorthy S., Minh B.Q., Wong T.K.F., von Haeseler A., Jermiin L.S. (2017). ModelFinder: Fast model selection for accurate phylogenetic estimates. Nat. Methods.

[29] Seemann T. (2019). Snp-Dists. GitHub. https://github.com/tseemann/snp-dists.

[30] Schürch A.C., Arredondo-Alonso S., Willems R.J.L., Goering R.V. (2018). Whole genome sequencing options for bacterial strain typing and epidemiologic analysis based on single nucleotide polymorphism versus gene-by-gene-based approaches. Clin. Microbiol. Infect..

[31] Gorrie C.L., Mirčeta M., Wick R.R., Judd L.M., Lam M.M.C., Gomi R., Abbott I.J., Thomson N.R., Strugnell R.A., Pratt N.F. (2022). Genomic dissection of *Klebsiella pneumoniae* infections in hospital patients reveals insights into an opportunistic pathogen. Nat. Commun..

[32] Romano-Bertrand S., Virieux-Petit M., Mauffrey F., Senn L., Blanc D.S. (2025). Defining a genomic threshold for investigating *Pseudomonas aeruginosa* hospital outbreak. J. Hosp. Infect..

[33] Cui X., Lin C., Zhu W., Zhang X., Wang W., Ye L., Liu W., Liu S. (2026). Whole-genome sequencing identifies persistent transmission of a high-risk ST2 *Acinetobacter baumannii* clone in a Guangzhou Hospital. Front. Microbiol..

[34] Rozwandowicz M., Brouwer M.S.M., Fischer J., Wagenaar J.A., Gonzalez-Zorn B., Guerra B., Mevius D.J., Hordijk J. (2018). Plasmids carrying antimicrobial resistance genes in Enterobacteriaceae. J. Antimicrob. Chemother..

[35] Rocha-Gracia R.D.C., Lozano-Zarain P., Cázarez Z.G., Alonso C.A., Brambila E., Torres C., Cortés-Cortés G. (2022). IncFIB plasmids carrying the resistance gene blaCTX-M-15 in ESBL-producing *Escherichia coli* clones from pediatric patients. J. Infect. Dev. Ctries..

[36] Johnson T.J., Nolan L.K. (2009). Pathogenomics of the virulence plasmids of *Escherichia coli*. Microbiol. Mol. Biol. Rev..

[37] Alhejaili A.Y., Zhou G., Halawa H., Huang J., Fallatah O., Hirayban R., Iftikhar S., AlAsmari A., Milner M., Banzhaf M. (2025). Methicillin-resistant *Staphylococcus aureus* in Saudi Arabia: Genomic evidence of recent clonal expansion and plasmid-driven resistance dissemination. Front. Microbiol..

[38] Schwarz S., Shen J., Kadlec K., Wang Y., Michael G.B., Feßler A.T., Vester B. (2016). Lincosamides, Streptogramins, Phenicols, and Pleuromutilins: Mode of Action and Mechanisms of Resistance. Cold Spring Harb. Perspect. Med..

[39] Xu W., Chen T., Wang H., Zeng W., Wu Q., Yu K., Xu Y., Zhang X., Zhou T. (2020). Molecular Mechanisms and Epidemiology of Fosfomycin Resistance in *Staphylococcus aureus* Isolated from Patients at a Teaching Hospital in China. Front. Microbiol..

[40] Umeda K., Nakamura H., Yamamoto K., Nishina N., Yasufuku K., Hirai Y., Hirayama T., Goto K., Haset A., Ogasawara J. (2017). Molecular and epidemiological characterization of staphylococcal foodborne outbreak of *Staphylococcus aureus* harboring *seg*, *sei*, *sem*, *sen*, *seo*, and *selu* genes without production of classical enterotoxins. Int. J. Food Microbiol..

[41] Mathers A.J., Peirano G., Pitout J.D. (2015). The role of epidemic resistance plasmids and international high-risk clones in the spread of Multidrug-Resistant Enterobacteriaceae. Clin. Microbiol. Rev..

[42] Carattoli A. (2013). Plasmids and the spread of resistance. Int. J. Med. Microbiol..

[43] Bi D., Zheng J., Li J., Sheng Z.-K., Zhu X., Ou H.-Y., Li Q., Wei Q. (2018). In Silico Typing and Comparative Genomic Analysis of IncFII K Plasmids and Insights into the Evolution of Replicons, Plasmid Backbones, and Resistance Determinant Profiles. Antimicrob. Agents Chemother..

[44] Cai M., Song K., Yao C., Wang S., Wang R., Wang Q., Chen H., Wang H. (2026). Global spread and evolution of KPC-2 and NDM-1-producing Gram-negative bacteria. Sci. China Life Sci..

[45] Mendoza-Guido B., Durán-Méndez S., Barrantes K., Rojas-Jimenez K., Chacón L. (2026). Genomic analysis of IncH plasmids reveals their role as drivers of antimicrobial resistance and adaptive traits in enterobacterales. FEMS Microbiol. Lett..

[46] Poirel L., Bonnin R.A., Nordmann P. (2012). Genetic features of the widespread plasmid coding for the carbapenemase OXA-48. Antimicrob. Agents Chemother..

[47] Osunla C.A., Akinbobola A., Elshafea A., Yeboah E.E.A., Bakare O.S., Fayanju A., Fatoba D.O., Boamah B., Amoako D.G. (2025). Genomic and bioinformatic insights into *Enterococcus faecalis* from retail meats in Nigeria. Int. J. Microbiol..

[48] Song W., Kim J., Bae I.K., Jeong S.H., Seo Y.H., Shin J.H., Jang S.J., Uh Y., Shin J.H., Lee M.-K. (2011). Chromosome-encoded AmpC and CTX-M extended-spectrum β-lactamases in clinical isolates of *Proteus mirabilis* from Korea. Antimicrob. Agents Chemother..

[49] Wand M.E., Bock L.J., Bonney L.C., Sutton J.M. (2017). Mechanisms of increased resistance to chlorhexidine and cross-resistance to colistin following exposure of *Klebsiella pneumoniae* clinical isolates to chlorhexidine. Antimicrob. Agents Chemother..

[50] Hamidian M., Nigro S.J. (2019). Emergence, molecular mechanisms and global spread of carbapenem-resistant *Acinetobacter baumannii*. Microb. Genom..

[51] Karah N., Sundsfjord A., Towner K., Samuelsen Ø. (2012). Insights into the global molecular epidemiology of carbapenem non-susceptible clones of *Acinetobacter baumannii*. Drug Resist. Updat..

[52] Mugnier P.D., Poirel L., Naas T., Nordmann P. (2010). Worldwide dissemination of the bla_OXA-23_ carbapenemase gene of *Acinetobacter baumannii*. Emerg. Infect. Dis..

[53] Karah N., Haldorsen B., Hegstad K., Simonsen G.S., Sundsfjord A., Samuelsen Ø. (2011). Species identification and molecular characterization of Acinetobacter spp. blood culture isolates from Norway. J. Antimicrob. Chemother..

[54] Tchesnokova V.L., Rechkina E., Chan D., Haile H.G., Larson L., Ferrier K., Schroeder D.W., Solyanik T., Shibuya S., Hansen K. (2020). Pandemic uropathogenic fluoroquinolone-resistant *Escherichia coli* have enhanced ability to persist in the gut and cause bacteriuria in healthy women. Clin. Infect. Dis..

[55] Pitout J.D.D., Peirano G., Chen L., DeVinney R., Matsumura Y. (2022). *Escherichia coli* ST1193: Following in the footsteps of *E. coli* ST131. Antimicrob. Agents Chemother..

[56] Zurita J., Sevillano G., Paz y Miño A., Haro N., Larrea-Álvarez M., Alcocer I., Ortega-Paredes D. (2023). Dominance of ST131, B2, blaCTX-M-15, and papA-papC-kpsMII-uitA among ESBL *Escherichia coli* isolated from bloodstream infections in Quito, Ecuador: A 10-year surveillance study (2009–2019). J. Appl. Microbiol..

[57] Turner N.A., Sharma-Kuinkel B.K., Maskarinec S.A., Eichenberger E.M., Shah P.P., Carugati M., Holland T.L., Fowler V.G. (2019). Methicillin-resistant *Staphylococcus aureus*: An overview of basic and clinical research. Nat. Rev. Microbiol..

[58] Sato T., Yamaguchi T., Aoki K., Kajiwara C., Kimura S., Maeda T., Yoshizawa S., Sasaki M., Murakami H., Hisatsune J. (2023). Whole-genome sequencing analysis of molecular epidemiology and silent transmissions causing meticillin-resistant *Staphylococcus aureus* bloodstream infections in a university hospital. J. Hosp. Infect..

[59] David M.Z., Daum R.S. (2010). Community-associated methicillin-resistant *Staphylococcus aureus*: Epidemiology and clinical consequences of an emerging epidemic. Clin. Microbiol. Rev..

[60] Feltrin F., Alba P., Kraushaar B., Ianzano A., Argudín M.A., Di Matteo P., Porrero M.C., Aarestrup F.M., Butaye P., Franco A. (2015). A livestock-associated, multidrug-resistant, methicillin-resistant *Staphylococcus aureus* clonal complex 97 lineage spreading in dairy cattle and pigs in Italy. Appl. Environ. Microbiol..

[61] Guan L., Beig M., Wang L., Navidifar T., Moradi S., Tabaei F.M., Teymouri Z., Moghaldam M.A., Sedighi M. (2024). Global status of antimicrobial resistance in clinical *Enterococcus faecalis* isolates: Systematic review and meta-analysis. Ann. Clin. Microbiol. Antimicrob..

[62] Coburn P.S., Glimore M.S. (2003). *Enterococcus faecalis* cytolysin: A novel toxin active against eukaryotic and prokaryotic cells. Cell. Microbiol..

[63] Top J., Willems R., Bonten M. (2007). Emergence of Clonal Complex 17 *Enterococcus faecium* in the Netherlands. J. Clin. Microbiol..

[64] Hegstad K., Mikalsen T., Coque T.M., Werner G., Sundsfjord A. (2010). Mobile genetic elements and their contribution to the emergence of antimicrobial-resistant *Enterococcus faecalis* and *Enterococcus faecium*. Clin. Microbiol. Infect..

[65] Zhang P., Cheng Z., Cao Y., Liu S., Zhao M. (2026). Genomic epidemiology of antimicrobial resistance in *Proteus mirabilis*: Core genome and plasmid-mediated drivers. BMC Microbiol..

[66] Wu J.J., Chen H.-M., Ko W.-C., Wu H.-M., Tsai S.-H., Yan J.-J. (2008). Prevalence of extended-spectrum β-lactamases in *Proteus mirabilis* in a Taiwanese university hospital, 1999 to 2005: Identification of a novel CTX-M enzyme (CTX-M-66). Diagn. Microbiol. Infect. Dis..

[67] Del Barrio-Tofiño E., López-Causapé C., Oliver A. (2020). *Pseudomonas aeruginosa* epidemic high-risk clones and their association with horizontally-acquired β-lactamases: 2020 update. Int. J. Antimicrob. Agents.

[68] Treepong P., Kos V., Guyeux C., Blanc D., Bertrand X., Valot B., Hocquet D. (2017). Global emergence of the widespread *Pseudomonas aeruginosa* ST235 clone. J. Clin. Microbiol. Infect..

[69] Takahashi T., Tada T., Shrestha S., Hishinuma T., Sherchan J.B., Tohya M., Kirikae T., Sherchand J.B. (2021). Molecular characterisation of carbapenem-resistant *Pseudomonas aeruginosa* clinical isolates in Nepal. J. Glob. Antimicrob. Resist..

[70] Botelho J., Grosso F., Peixe L. (2019). Antibiotic resistance in *Pseudomonas aeruginosa*—Mechanisms, epidemiology and evolution. Drug Resist. Updat..

[71] Otter J.A., Yezli S., French G.L. (2011). The role played by contaminated surfaces in the transmission of nosocomial pathogens. Infect. Control Hosp. Epidemiol..

[72] de Bastiani D.C., Silva C.V., Christoff A.P., Cruz G.N.F., Tavares L.D., de Araújo L.S.R., Tomazini B.M., Arns B., Piastrelli F.T., Cavalcanti A.B. (2024). 16S rRNA amplicon sequencing and antimicrobial resistance profile of intensive care units environment in 41 Brazilian hospitals. Front. Public Health.

[73] Zhang G., Zhou Y., Yan B., Wu C., Yang Y., Qiu T., Wu J., Wen S., Chen Y., Deng X. (2025). The evolution of the multidrug-resistant and globally distributed ST2 *Acinetobacter baumannii* in China. BMC Microbiol..

[74] Duan Z., Li X., Li S., Zhou H., Hu L., Xia H., Xie L., Xie F. (2024). Nosocomial surveillance of multidrug-resistant *Acinetobacter baumannii*: A genomic epidemiological study. Microbiol. Spectr..

[75] Fox J.M., Saunders N.J., Jerwood S.H. (2023). Economic and health impact modelling of a whole genome sequencing-led intervention strategy for bacterial healthcare-associated infections for England and for the USA. Microb. Genom..

[76] Pustijanac E., Hrenović J., Vranić-Ladavac M., Močenić M., Karčić N., Stefanović L.L., Hrstić I., Lončarić J., Musić M.Š., Drčelić M. (2023). Dissemination of clinical *Acinetobacter baumannii* isolate to hospital environment during the COVID-19 pandemic. Pathogens.

[77] Bedenić B., Bratić V., Mihaljević S., Lukić A., Vidović K., Reiner K., Schöenthaler S., Barišić I., Zarfel G., Grisold A. (2023). Multidrug-resistant bacteria in a COVID-19 hospital in Zagreb. Pathogens.

[78] Zingg S., Kuster S., von Rotz M., Portmann A., Egli A., Seth-Smith H.M., Schlaepfer P., Goldenberger D., Bassetti S., Marsch S. (2024). Outbreak with OXA-23-producing *Acinetobacter baumannii* in a COVID-19 ICU cohort: Unraveling routes of transmission. Antimicrob. Resist. Infect. Control.

[79] Cureño-Díaz M.A., Plascencia-Nieto E.S., Loyola-Cruz M.Á., Cruz-Cruz C., Nolasco-Rojas A.E., Durán-Manuel E.M., Ibáñez-Cervantes G., Gómez-Zamora E., Tamayo-Ordóñez M.C., Tamayo-Ordóñez Y.D.J. (2024). Gram-negative ESKAPE bacteria surveillance in COVID-19 pandemic exposes high-risk sequence types of *Acinetobacter baumannii* MDR in a tertiary care hospital. Pathogens.

